# Overexpression of OsPUB41, a Rice E3 ubiquitin ligase induced by cell wall degrading enzymes, enhances immune responses in Rice and Arabidopsis

**DOI:** 10.1186/s12870-019-2079-1

**Published:** 2019-11-29

**Authors:** Neha Rajendra Kachewar, Vishal Gupta, Ashish Ranjan, Hitendra Kumar Patel, Ramesh V. Sonti

**Affiliations:** 1 CSIR-Centre for Cellular and Molecular Biology, Uppal Road, Hyderabad, 500007 India; 2 National Institute of Plant Genome Research, New Delhi, 110067 India; 3 Department of Plant Pathology, University of Wisconsin-Madison, Madison, WI 53706 USA

**Keywords:** Cell wall degrading enzymes, Damage associated molecular patterns, E3 ubiquitin ligase, Xoo, OsPUB41, Plant immunity and *Rhizoctonia solani*

## Abstract

**Background:**

Cell wall degrading enzymes (CWDEs) induce plant immune responses and E3 ubiquitin ligases are known to play important roles in regulating plant defenses. Expression of the rice E3 ubiquitin ligase, *OsPUB41,* is enhanced upon treatment of leaves with *Xanthomonas oryzae* pv. *oryzae* (Xoo) secreted CWDEs such as Cellulase and Lipase/Esterase. However, it is not reported to have a role in elicitation of immune responses.

**Results:**

Expression of the rice E3 ubiquitin ligase, *OsPUB41,* is induced when rice leaves are treated with either CWDEs, pathogen associated molecular patterns (PAMPs), damage associated molecular patterns (DAMPs) or pathogens. Overexpression of *OsPUB41* leads to induction of callose deposition, enhanced tolerance to Xoo and *Rhizoctonia solani* infection in rice and Arabidopsis respectively. In rice, transient overexpression of *OsPUB41* leads to enhanced expression of *PR* genes and SA as well as JA biosynthetic and response genes. However, in Arabidopsis, ectopic expression of *OsPUB41* results in upregulation of only JA biosynthetic and response genes. Transient overexpression of either of the two biochemically inactive mutants (*OsPUB41C40A* and *OsPUB41V51R*) of *OsPUB41* in rice and stable transgenics in Arabidopsis ectopically expressing *OsPUB41C40A* failed to elicit immune responses. This indicates that the E3 ligase activity of OsPUB41 protein is essential for induction of plant defense responses.

**Conclusion:**

The results presented here suggest that OsPUB41 is possibly involved in elicitation of CWDE triggered immune responses in rice.

## Background

Plants have evolved very intricate and complex systems to cope with microbial infection. One of them is PAMP-triggered immunity (PTI), which is induced upon recognition of either conserved microbial molecules called pathogen-associated molecular patterns (PAMPs) or their own molecules released due to damage caused by the pathogen called damage-associated molecular patterns (DAMPs). Activation of PTI leads to various responses like callose deposition, production of reactive oxygen species, expression of defense genes, etc. [1].

Cell wall degrading enzymes (CWDEs) secreted by microbial pathogens have been long known to elicit plant defense responses such as production of phytoalexins, oxidative burst, strengthening of cell wall, etc. [2]. Endopolygalacturonic acid lyase, purified from *Erwinia carotovora* culture filtrates, has been shown to release oligosaccharides from soybean cell walls and thereby trigger phytoalexin accumulation in soybean [3]. Prior treatment of tobacco seedlings with CWDEs like pectate lyase or polygalacturonase from *Erwinia carotovora* subsp. *carotovora* induced resistance against subsequent *E. c.* subsp. *carotovora* infection [4]. However, the molecules involved in regulation of CWDEs induced plant innate immunity are not well studied.

*Xanthomonas oryzae* pv. *oryzae* (Xoo), the bacterial blight pathogen of rice, secretes a battery of cell wall degrading enzymes (CWDE) such as lipase/esterase (LipA), cellulase (ClsA), xylanase (XynB), and cellobiosidase (CbsA) [5, 6]. Although they are important for virulence, these enzymes are double-edged swords as they induce rice defense responses such as callose deposition and programmed cell death. Also, prior treatment of rice leaves with any of these enzymes results in enhanced tolerance to subsequent Xoo infection [5]. It appears that CWDEs such as LipA act upon rice cell walls and release degradation products that act as DAMPs and elicit defense responses. In order to identify rice functions that may be involved in CWDE-induced defense responses, we had performed transcriptome analyses following treatment of rice leaves with purified ClsA [7] (12 h post-treatment with the enzyme) and LipA [12 h time-point [8] and 2 h time-point: GEO-ID: GSE53940]. In all of these analyses, the expression of *OsPUB41*, an E3 ubiquitin ligase gene (Class III U-box type), was found to be enhanced. In addition, at 2 h time-point *OsPUB41* was the only E3 ligase, whose expression was significantly induced (> 1.5 fold up and *p* value ≤0.05) at this time point amongst 77 U-box genes annotated in rice genome. E3 ligases are known to be involved in regulating plant innate immune responses [9–12]. Earlier reports have indicated that the predicted protein contains an N-terminal U-box domain (~ 70 amino acids) and a conserved GKL domain (~ 100 amino acids with conserved glycine [G] and lysine [K]/arginine [R] residues as well as a leucine [L]-rich feature) near the C-terminus [11, 13]. OsPUB41 has been shown to be an active polyubiquitinating E3 ligase [13]. Here we report that transient overexpression of *OsPUB41* in rice leaves results in induction of callose deposition and enhanced resistance against subsequent Xoo infection. Transient overexpression of *OsPUB41* in rice induces expression of various JA and SA biosynthetic and response genes along with a set of *Pathogenesis Related* genes. Stable transgenics in Arabidopsis ectopically expressing *OsPUB41* exhibited enhanced callose deposition, expression of various JA biosynthetic as well as response genes and enhanced tolerance to *Rhizoctonia solani* AGI-1A (*R. solani*) infection. In addition, overexpression of biochemically inactive OsPUB41 mutants (OsPUB41C40A and OsPUB41V51R) failed to elicit immune responses in rice and Arabidopsis. These results indicate that OsPUB41 might be a positive regulator of innate immunity and that the biochemical activity of OsPUB41 is necessary for elicitation of defense responses.

## Results

### Enhanced expression of *OsPUB41* was observed following treatment with either CWDEs, elicitors or pathogens

Previously, microarray analyses had been performed following treatment of rice leaves with either purified LipA [12 h time point [8] and 2 h time point, GEO-ID: GSE53940] or ClsA [7]. *OsPUB41* was the only E3 ubiquitin ligase gene that was found to be upregulated in all of these treatments (Table 1). In addition, expression of *OsPUB41* was found to be enhanced when rice leaves were infiltrated with commercially available CWDEs such as fungal cellulase, pectinase and xylanase.

**Table 1:** Expression of *OsPUB41* is induced following treatment of rice leaves with various cell wall degrading enzymes

Experimental parameters	Microarray data ^b^			qPCR data ^c^		
CWDEs^a^	Lipase A (from Xoo)		Cellulase A (from Xoo)	Cellulase (from *Trichoderma viride*)	Xylanase (from *Trichoderma viride*)	Pectinase (from *Aspergillus niger*)
Time point	2 h	12 h	12 h	12 h	12 h	12 h
Fold change in *OsPUB41* expression	3.15	10.4	2.8	4.3 ± 1.16	2.9 ± 0.1	11 ± 4.6

^a^Leaves of ten-fifteen-days-old Taichung Native-1 (TN-1) rice plants were pressure infiltrated, using a needleless syringe, with any one of the bacterial or fungal CWDEs

^b^Relative fold change of *OsPUB41* in microarray analysis observed using either Lipase A (GEO-ID: GSE53940, GSE49242) or Cellulase A (GSE8216) (*p* < 0.05)

^c^Relative fold change of *OsPUB41* (average value from three independent experiments, ± represents standard error) when treated with CWDEs as compared to mock treatment. *OsActin* was used as an internal control in qPCR for rice. Student’s two-tailed t-test for independent means was performed on delta C_t_ values to test for significance (*p* < 0.05)

Interestingly, *OsPUB41* expression was also induced upon treating rice with either DAMPs (eATP and sucrose) or PAMPs (Flg22 or LPS) (Additional file 1: Table S1). In addition, analysis of publicly available microarray data from GEO database revealed that the expression of *OsPUB41* is enhanced when rice is infected by either a fungal (*Magnaporthe grisea* FR13 and *Magnaporthe oryzae* Guy11) or a bacterial (Xoo strains: PXO99A and PXO86) (Additional file 2: Table S2) pathogen. In addition, treatment of TN1 rice leaves with Xoo (strain BXO43; that we used in the experiment) also induced expression of *OsPUB41* (Additional file 2: Table S2).

We analysed the OsPUB41 protein sequence using InterPro tool [14] which suggested that OsPUB41 has an N- terminal U-box domain (amino acid position 33 to 112) and an Armadillo-type fold (amino acid position 141–435) (Additional file 3: Fig. S1A, S1B) which are known to mediate ubiquitination and protein-protein interactions, respectively.

### Transient overexpression of *OsPUB41* in rice leaves results in elicitation of callose deposition and enhanced tolerance to infection by Xoo

**Fig. 1:**
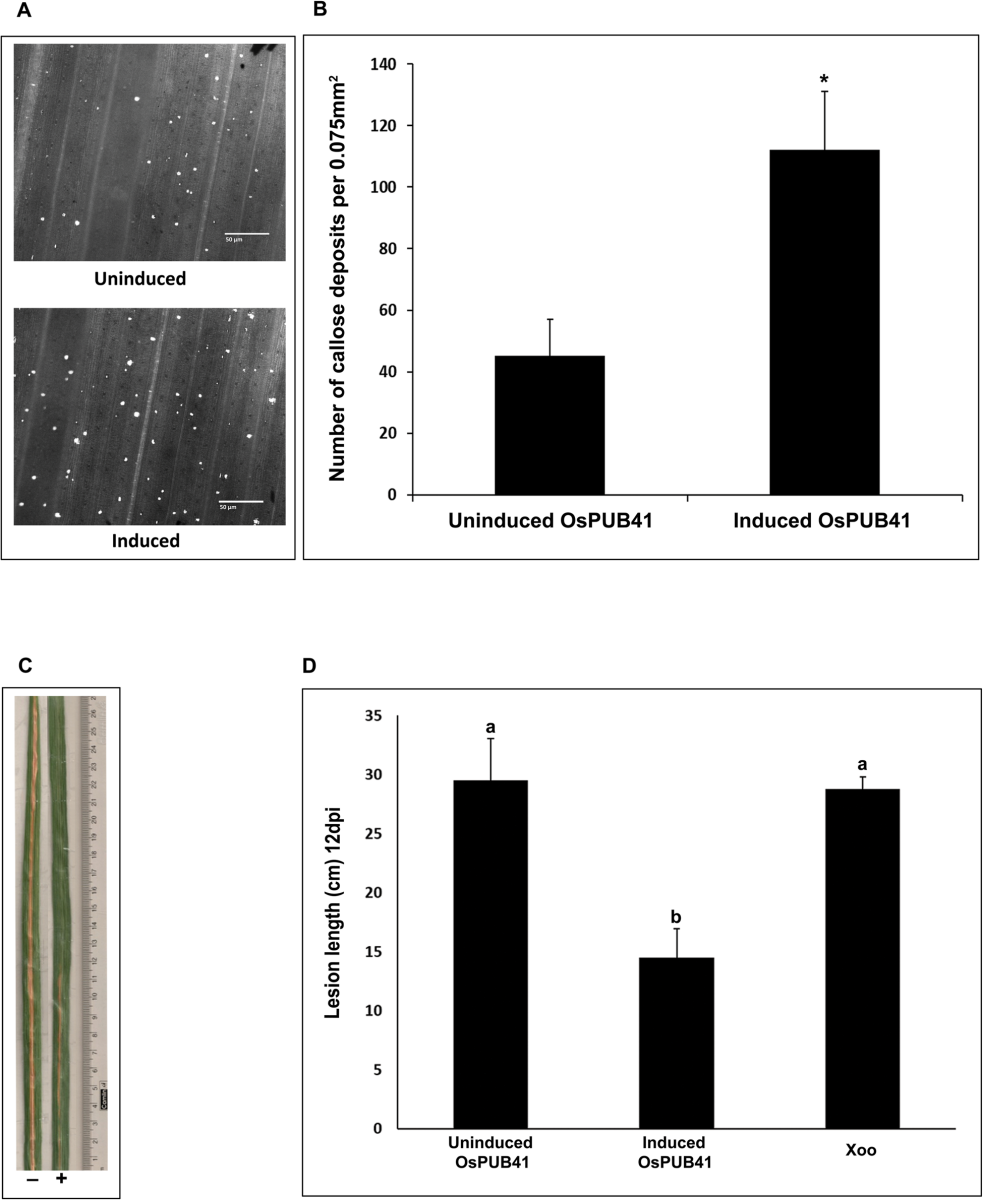
Overexpression of *OsPUB41* induces callose deposition and provides enhanced tolerance to Xoo infection in rice. Rice leaves were infiltrated with *Agrobacterium* LBA4404/pMDC7-OsPUB41 strain either with inducer (estradiol) or with DMSO (control). Twelve hours later, the leaves were stained with aniline blue and observed under an epifluorescence microscope. Bright spots in the images represent callose deposits (**a**). Scale bar represents 50 μm. The graph represents average number of callose deposits per field of view (0.075 mm^2^) from atleast ten leaves with six to eight different fields viewed per leaf in each experiment (**b**). Error bars represent standard error. Student’s two-tailed t-test for independent means was performed to test for significance (*p* < 0.05, represented by*). Similar results were obtained in three independent experiments. **c**. Xoo infections were carried out in midveins of leaves (*n* = 20–25) of 40-days-old TN-1 rice plants. The midveins were pre-injected with LBA4404/pMDC7-OsPUB41 with or without estradiol. After 12 h, these midveins were inoculated with Xoo (1–2 cm below the point where *Agrobacterium* was injected) by pricking with a needle dipped in a saturated Xoo culture. Yellowing represents bacterial blight lesions that were observed 12dpi. The graph represents average lesion lengths from at least twenty leaves in each experiment (**d**). Error bars represent standard error. Student’s two-tailed t-test for independent means was performed to test for significance (*p* < 0.05, ‘a’ and ‘b’ represent statistically different values). Similar results were obtained in three independent experiments

Callose deposition is a marker of plant innate immune response. Purified preparations of CWDEs such as LipA, ClsA or CbsA induce callose deposition in rice leaves [5, 15]. *OsPUB41* was transiently overexpressed in rice and the effect on callose deposition was assessed. For transient overexpression, an estradiol-inducible construct was used [16]. In the presence of inducer (Estradiol), the expression of *OsPUB41* was induced 12–13 fold as compared to the uninduced state (DMSO) (Additional file 4: Fig. S2A). Immunoblotting was also performed to confirm expression of *OsPUB41* in rice leaves (Additional file 4: Fig. S2B). Under conditions of *OsPUB41* overexpression, there is a significant increase in the number of callose deposits (Fig. 1a, b). Estradiol by itself does not induce callose deposition in rice (Additional file 5: Table. S3).

Prior treatment of rice leaves with a CWDE such as LipA or ClsA results in enhanced tolerance to subsequent Xoo infection [5]. Therefore, we checked whether overexpression of *OsPUB41* would result in enhanced tolerance to Xoo infection. For this, midveins of leaves of 40–45-days-old rice plants were injected with *Agrobacterium* containing an estradiol-inducible *OsPUB41* overexpression construct in the presence (induced) or absence (uninduced) of estradiol. Twelve hours later, these plants were infected with Xoo by pricking their midveins with a needle touched to a pellet of saturated Xoo culture. Lesion lengths were measured 12 days after infection. Under conditions of *OsPUB41* expression, the lesion lengths were about 14 cm long while the lesion lengths were about 29 cm long in the absence of *OsPUB41* overexpression. Similar size lesions (approximately 29 cm long) were observed in rice leaves that had been treated with Xoo without any prior treatment with *Agrobacterium* (Fig. 1c, d). Thus, overexpression of *OsPUB41* leads to enhanced tolerance against subsequent Xoo infection in rice. Estradiol by itself does not affect Xoo infection in rice (Additional file 6: Table S4).

### Transient overexpression of *OsPUB41* results in enhanced expression of Rice defense genes

**Fig. 2:**
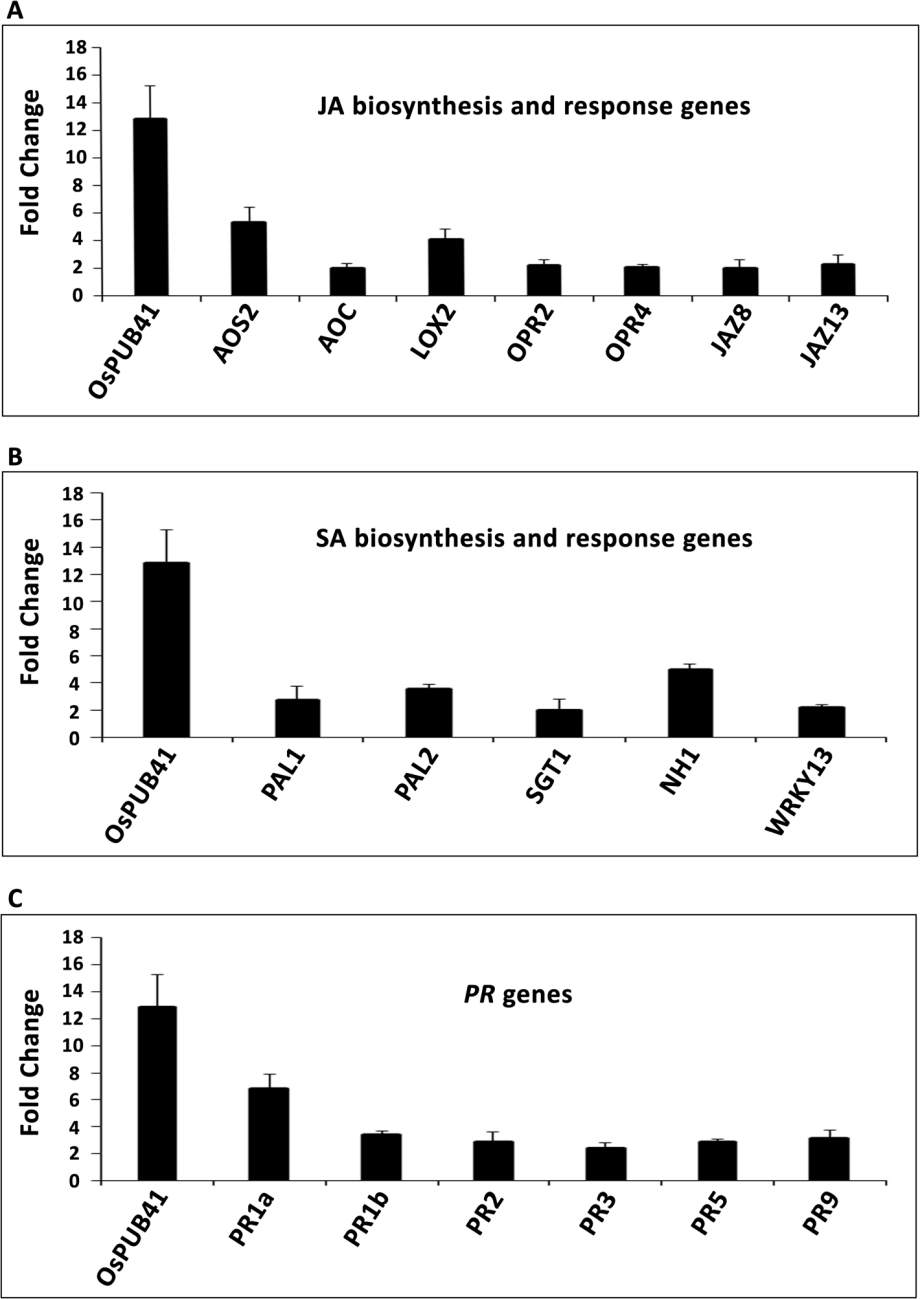
Transient overexpression of *OsPUB41* leads to enhanced expression of rice defense genes. Transcript levels of JA (**a**) and SA (**b**) biosynthetic and response genes and of *PR* genes (**c**), were measured by qPCR upon overexpression of *OsPUB41* in rice. *AOS2*: Allene oxide synthase2, *AOC*: Allene oxide cyclase, *LOX2*: Lipoxygenase2 and *OPR2* and *OPR4*: 12-oxophytodienoate reductase2 and 4 (JA biosynthetic genes), *JAZ8* and *JAZ13*: Jasmonate ZIM-Domain8 and 13 (JA response genes), *PAL1* and *PAL2*: Phenylalanine ammonia lyase1 and 2 (SA biosynthetic genes), *SGT1*: SA glucosyltransferase1, *NH1*: Non-expresser of PR1 homolog1 (SA response genes), *WRKY13*: SA and JA response gene. *OsActin* was used as an internal control in qPCR. The graph represents average fold change values from three biological replicates. Student’s two-tailed t-test for independent means was performed on delta C_t_ values to test for significance (*p* < 0.05)

A number of JA biosynthetic and response genes were found to be upregulated 12 h post treatment of rice leaves with purified CWDEs like ClsA [7] or LipA [8]. Treatment with LipA also led to an increase in the levels of (+)-7-iso-Jasmonoyl-L-isoleucine (JA-Ile), the bioactive form of JA [8]. We wanted to know if overexpression of *OsPUB41* would lead to an increase in the level of expression of genes associated with either JA biosynthesis or response. Similar (to LipA or ClsA treated rice leaves) fold changes or enhancements in expression of JA biosynthetic as well as response genes were observed upon transient overexpression of *OsPUB41* in rice leaves (Fig. 2a). We also found that overexpression of *OsPUB41* induced the expression of genes associated with SA biosynthesis and response (Fig. 2b). Expression of rice defense genes like *PR1a*, *PR1b*, *PR2*, *PR3*, *PR5* and *PR9* was also induced by overexpression of *OsPUB41* (Fig. 2c). Estradiol by itself neither affects expression of *PR* genes nor that of biosynthetic and response genes of JA and SA (Additional file 7: Table S5).

### Ectopic expression of *OsPUB41* results in induction of callose deposition in transgenic Arabidopsis lines

**Fig. 3:**
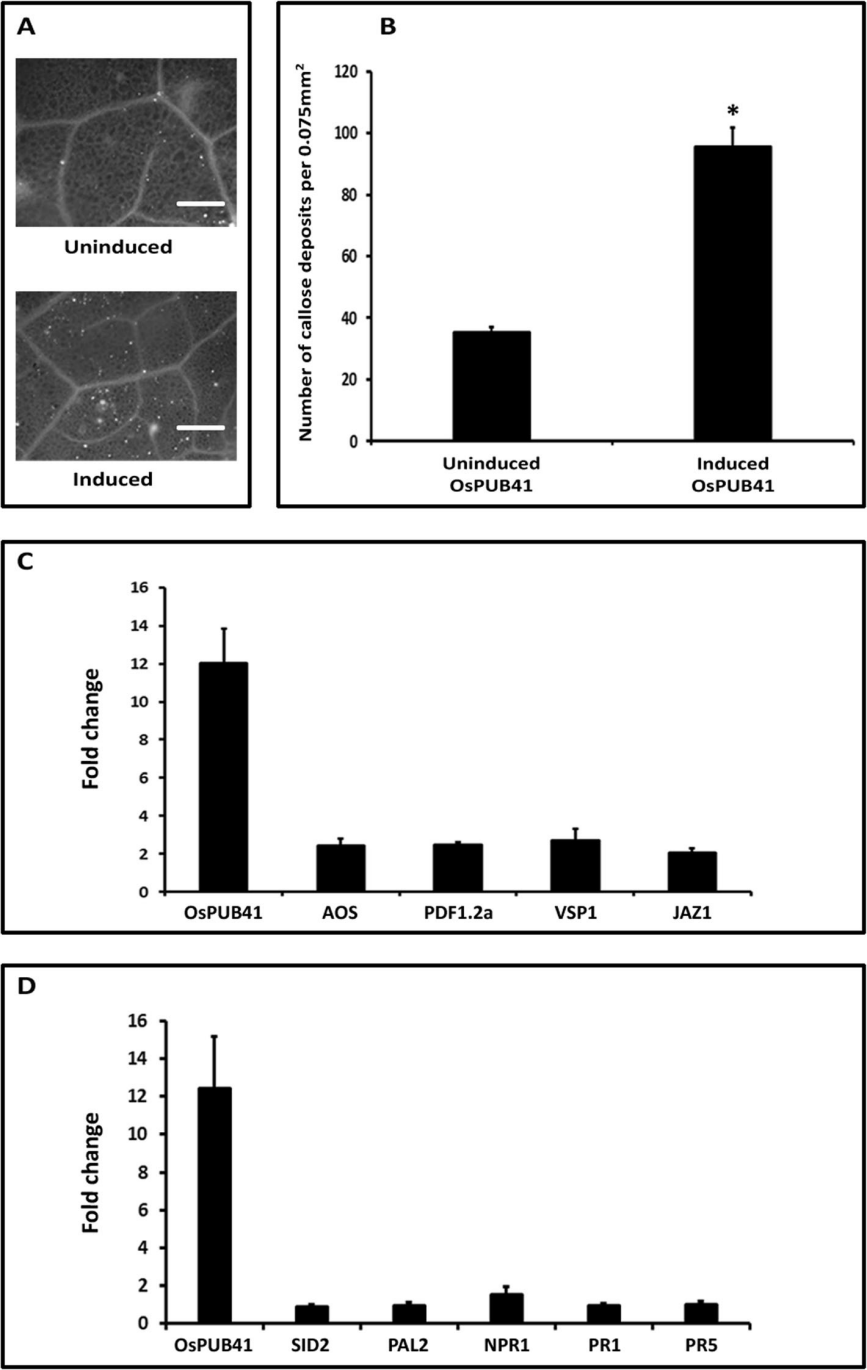
Ectopic expression of *OsPUB41* induces callose deposition, leads to enhanced expression of JA biosynthesis and response genes, but does not affect expression of SA biosynthesis and response genes in transgenic Arabidopsis lines. Leaves of thirty-days-old *OsPUB41* transgenic Arabidopsis plants were infiltrated either with estradiol (inducer) or with DMSO (uninduced). After 12 h, leaves were stained with aniline blue and observed under an epifluorescence microscope. Bright spots in the images represent callose deposits (**a**). Scale bar represents 50 μm. The graph represents average number of callose deposits per field of view (0.075 mm^2^) from five-six leaves with ten different fields viewed per leaf in each experiment (**b**). Error bars represent standard error. Student’s two-tailed t-test for independent means was performed to test for significance (*p* < 0.05, represented by*). Similar results were obtained in three independent experiments (per transgenic line) and in three independent transgenic lines. Transcript levels of JA (**c**) and SA (**d**), biosynthetic and response genes, were measured upon ectopic expression of *OsPUB41* in Arabidopsis. *AOS*: Allene Oxide Synthase (JA biosynthetic gene), *PDF1.2a*: Plant Defensin, *VSP*: Vegetative Storage Protein and *JAZ*: Jasmonate ZIM-Domain (JA response genes). *SID2*: SA Induction-Deficient 2 (SA biosynthetic gene), *PAL2*: Phenylalanine Ammonia-Lyase 2 (SA biosynthetic gene), *NPR1*: Nonexpresser of *PR1* (SA response gene), *PR1*: Pathogenesis Related gene 1 (SA response gene) and *PR5*: Pathogenesis Related gene 5 (SA response gene). *AtUbq5* was used as an internal control in qPCR. Three biological repeats were performed for each independent transgenic line. Similar results were obtained in three independent transgenic lines. Student’s two-tailed t-test for independent means was performed on delta C_t_ values to test for significance (*p* < 0.05)

Transgenic Arabidopsis plants that exhibit estradiol-inducible expression of *OsPUB41* were generated. The expression of *OsPUB41* was induced ~ 12 fold when the inducer (estradiol) was infiltrated into Arabidopsis leaves in comparison to the uninduced state (Additional file 8: Fig. S3). Induction of expression of *OsPUB41* led to a significant increase in the number of callose deposits (Fig. 3a, b). Similar results were obtained in three transgenic Arabidopsis lines ectopically expressing *OsPUB41* (Additional file 9: Table. S6). Estradiol by itself does not induce callose deposition (Additional file 5: Table S3). Hence, as observed in rice, expression of *OsPUB41* enhances callose deposition in Arabidopsis.

### Ectopic expression of *OsPUB41* results in enhanced expression of Arabidopsis genes involved in JA biosynthesis and response

Treatment with either cellulase (Sigma) or exposure to a DAMP like AtPEP1 has been reported to induce the expression of JA biosynthetic and response genes [17, 18]. A number of JA and SA biosynthetic and response genes were found to be upregulated upon transient overexpression of *OsPUB41* in rice. We wanted to know if ectopic expression of *OsPUB41* leads to an increase in the level of expression of Arabidopsis genes associated with JA and SA biosynthesis or response. An increased expression of JA markers, biosynthetic as well as response genes was observed in a transgenic Arabidopsis line ectopically expressing *OsPUB41* in an estradiol-inducible manner (Fig. 3c). A similar trend was observed in three independent transgenic Arabidopsis lines (Additional file 10: Table S7). In addition, the fold changes or enhancements observed in JA biosynthetic and response genes upon *OsPUB41* expression in Arabidopsis were comparable to those observed upon treating Arabidopsis leaves with either cellulase (Sigma) or AtPEP1. Ectopic expression of *OsPUB41* did not affect the expression level of genes associated with either SA biosynthesis or response (Fig. 3d). Similar results were observed in three independent transgenic Arabidopsis lines (Additional file 10: Table S7). Estradiol (by itself) does not affect expression of JA or SA biosynthetic and response genes in Arabidopsis (Additional file 7: Table S5).

### Ectopic expression of *OsPUB41* results in enhanced tolerance to *Rhizoctonia solani* AG1-IA infection in Arabidopsis

Overexpression of *OsPUB41* provides enhanced tolerance to subsequent Xoo infection in rice. We wanted to know whether ectopic expression of *OsPUB41* would provide enhanced tolerance to microbial infection in Arabidopsis. Transgenic Arabidopsis lines expressing *OsPUB41* were infected with either *Rhizoctonia solani* AG1-IA (*R. solani*, a fungal necroptroph) or *Pseudomonas syringae* pv. *tomato* DC3000 (Pst, a bacterial hemibiotroph).

**Fig. 4:**
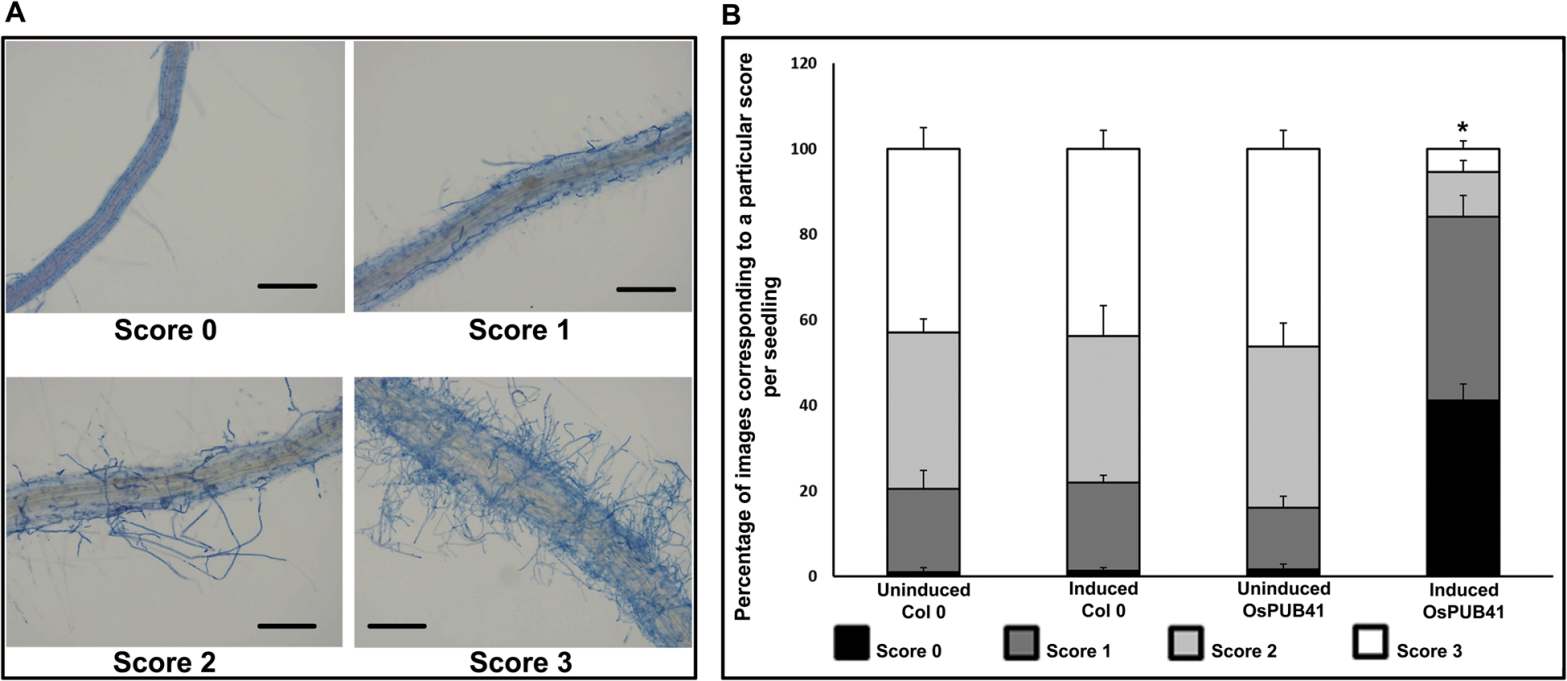
Ectopic expression of *OsPUB41* results in enhanced tolerance to *R. solani* infection in Arabidopsis. Fifteen-days-old Arabidopsis seedlings (Col-0 and *OsPUB41* transgenics) were infected with *R. solani*, in either the presence or absence of the inducer (Estradiol) of *OsPUB41* expression. Seven-days-post infection, the seedlings were stained with Trypan Blue and imaged using a light microscope. A scale with scores ranging from 0 to 3 was used to assess the extent of fungal infection. Score 0 = no hyphae, 1 = few unconnected hyphae, 2 = sparse continuous network of hyphae and 3 = dense network of hyphae (**a**). Scale bar represents 50 μm. The graph represents the frequency (as a percentage) of a particular score from ten seedlings with forty different fields viewed per seedling in each experiment (**b**). Scores of 0, 1, 2 and 3 are represented in the graph by black, dark grey, light grey and white respectively. Similar results were observed in three independent experiments (per transgenic line) and in three independent transgenic lines. One-way ANOVA was used to test for significance, followed by Tukey-Kramer honestly significant difference test (*p* < 0.05, represented by*)

In the transgenic *OsPUB41* Arabidopsis lines, induction of expression of *OsPUB41* (by treatment with estradiol) led to enhanced tolerance to *R. solani* infection (Fig. 4a, b) as compared to uninduced control plants. As an additional control, wild type Arabidopsis (ecotype Col-0) was used (with and without estradiol) in the infection assay to rule out the possibility that estradiol could have an effect on the infection process. A scoring scale of zero to three based on fungal load was used for assessing the extent and severity of *R. solani* infection with zero indicating a high level of tolerance and three indicating a high level of susceptibility. A majority of wild type Arabidopsis plants (with and without estradiol) and the *OsPUB41* transgenic plants (without inducer) exhibited scores of two or three. In contrast, a majority of *OsPUB41* transgenic plants in which the expression of *OsPUB41* had been induced exhibited a score of zero or one. Similar results were obtained in additional transgenic lines (Additional file 11: Table S8). Apart from qualitative analysis, the relative expression level of a fungal gene [18-28S ribosomal (r)DNA] as compared to a plant gene (*AtUbq5*) between induced (Estradiol) and uninduced (DMSO) samples indicated significantly less fungal load in *OsPUB41* expressing lines. (Value ~ 1 indicates similar fungal load whereas a value < 1, indicates less fungal load) (Additional file 12: Fig. S4). Similar results were obtained in additional transgenic lines (Additional file 13: Table S9). This shows that *OsPUB41* expression leads to enhanced tolerance to *R. solani* infection in Arabidopsis.

Bacterial counts obtained from growth yield assays performed in Col-0 and transgenic Arabidopsis plants (with or without induction), post Pst infection were similar (*p* value > 0.05) (Additional file 14: Fig. S5). Similar results were obtained in additional transgenic lines (Additional file 15: Table S10). Expression of *OsPUB41* had no significant effect on Pst infection in Arabidopsis.

### The C40A and V51R mutations of OsPUB41 affect the ability of the protein to elicit callose deposition and tolerance to Xoo infection in rice

**Fig. 5:**
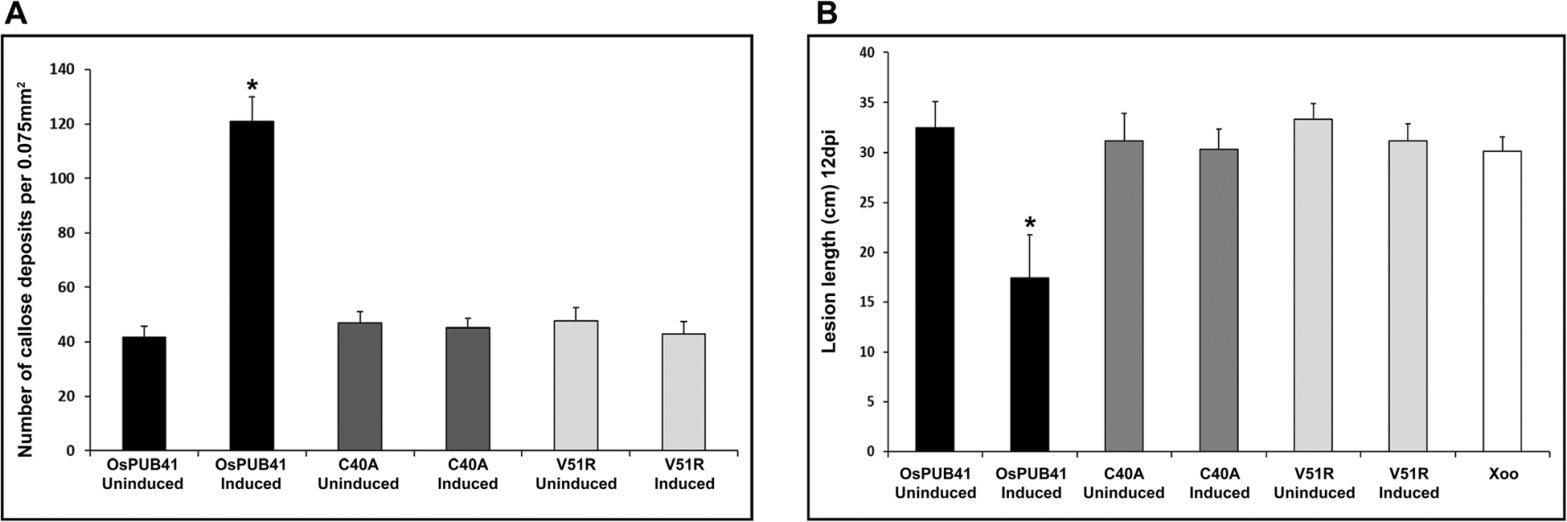
OsPUB41 mutant forms are incapable of inducing callose deposition and tolerance to Xoo infection in rice. Callose deposition was assayed upon transient overexpression of either *OsPUB41* or *OsPUB41C40A* or *OsPUB41V51R*. The graph represents average number of callose deposits per field of view (0.075 mm^2^) from ten leaves with six to eight different fields viewed per leaf in each experiment (**a**). Error bars represent standard error. Student’s two-tailed t-test for independent means was performed to test for significance (p < 0.05, represented by*). Similar results were obtained in three independent experiments. C40A and V51R represent *OsPUB41C40A* and *OsPUB41V51R* respectively. **b**. The bar represents average bacterial blight lesion length. Error bar represents standard error. Data was analyzed using the Student’s t-test for independent means (*indicates significant difference with *p* value < 0.05). Similar results were obtained in three independent experiments. C40A and V51R labels represent *OsPUB41C40A* and *OsPUB41V51R* respectively. Xoo label represents lesion lengths for leaves with Xoo infection without any prior treatment of *Agrobacterium*

OsPUB41 mutant forms (*OsPUB41C40A* or *OsPUB41V51R*) were bacterially expressed, purified (Additional file 16: Fig. S6) and found to be biochemically inactive in an in vitro auto-ubiquitination assay in which OsPUB41 was active (Additional file 17: Fig. S7). Expression levels of *OsPUB41* mutants (*OsPUB41C40A* or *OsPUB41V51R*) upon transient overexpression, as confirmed by qPCR (Additional file 4: Fig. S2A) and immunoblotting (Additional file 4: Fig. S2B) were found to be similar to that of *OsPUB41*. In *Agrobacterium* mediated transient transfer assays, induction of expression of the *OsPUB41* mutants did not result in enhanced callose deposition in rice leaves (Fig. 5a). In contrast, induction of expression of wild type *OsPUB41* led to enhanced callose deposition. Induction of expression of either *OsPUB41C40A* or *OsPUB41V51R* mutants did not result in enhanced tolerance to Xoo infection in rice leaves. Lesions of similar lengths were produced, irrespective of whether expression of genes encoding *OsPUB41C40A* and *OsPUB41V51R* mutations were induced or not induced (Fig. 5b). In contrast, induction of expression of wild type *OsPUB41* gene led to reduction in lesions caused by Xoo.

### The C40A mutation of OsPUB41 affects the ability of the protein to elicit callose deposition and tolerance to *R. solani* infection in Arabidopsis

**Fig. 6:**
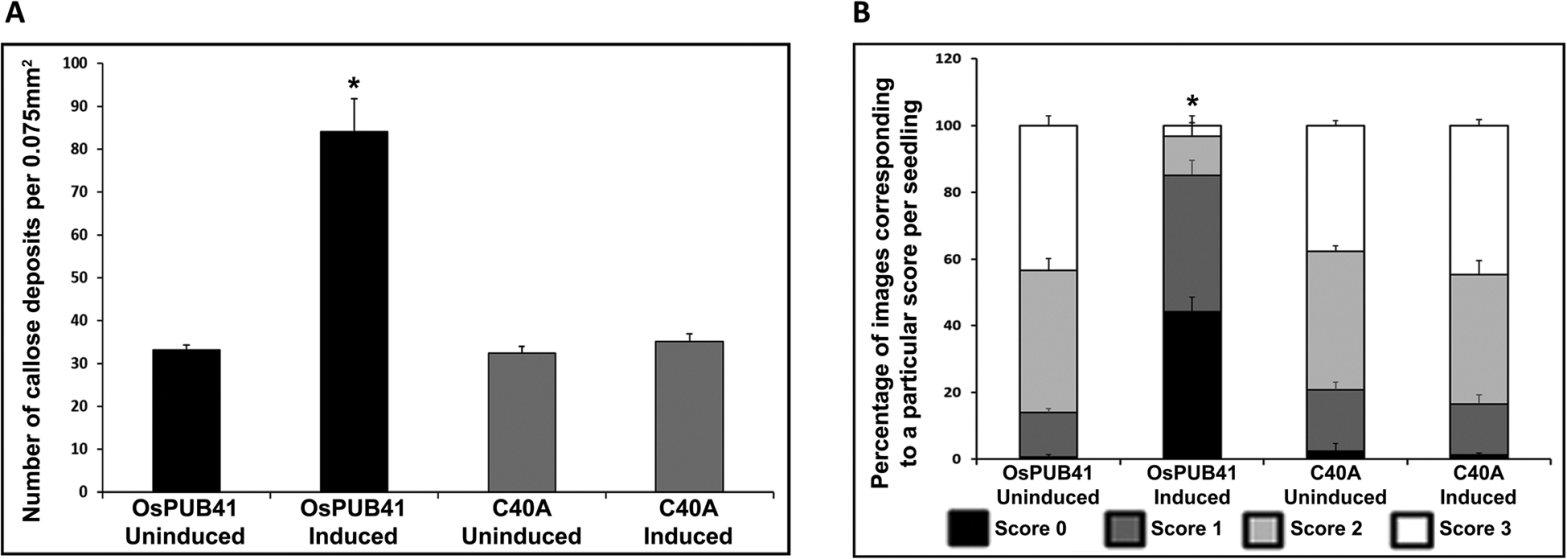
OsPUB41C40A is incapable of eliciting callose deposition and tolerance to *R. solani* infection in Arabidopsis. The graph represents average number of callose deposits per field of view (0.075 mm^2^) from five-six leaves with ten different fields viewed per leaf in each experiment (**a**). Error bars represent standard error. Student’s two-tailed t-test for independent means was performed to test for significance (p < 0.05, represented by*). Similar results were obtained in three independent experiments with three independent transgenic lines. **b**. Transgenic Arabidopsis seedlings (carrying *OsPUB41* or *OsPUB41C40A*) were infected with *R. solani*, either in the presence or absence of the inducer (Estradiol) of transgene expression. Infected seedlings were stained with Trypan Blue and imaged using a light microscope. A scale with scores ranging from 0 to 3 was used to assess the extent of fungal infection. Score 0 = no infection/hyphae, 1 = few unconnected hyphae, 2 = sparse continuous network of hyphae and 3 = dense network of hyphae. The graph represents the frequency (as a percentage) of a particular score from ten seedlings with forty different fields viewed per seedling in each experiment. Scores of 0, 1, 2 and 3 are represented in the graph by black, dark grey, light grey and white respectively. Seedlings from three independent transgenic lines were used for each experiment. A similar trend was observed in three independent experiments. One-way ANOVA was used to test for significance, followed by Tukey-Kramer honestly significant difference test (p < 0.05, represented by*). C40A label represents OsPUB41C40A

Transgenic Arabidopsis plants that exhibit estradiol-inducible expression of *OsPUB41C40A* were generated. Expression level of *OsPUB41C40A* was found to be similar to that of *OsPUB41* upon induction in transgenic Arabidopsis plants (Additional file 8: Fig. S3). Unlike transgenic plants expressing wild type *OsPUB41*, no enhancement in amount of callose deposition was observed in Arabidopsis transgenic lines expressing *OsPUB41C40A* (Fig. 6a). Similar results were obtained in additional transgenic lines ectopically expressing either *OsPUB41* or *OsPUB41C40A* (Additional file 9: Table S6). Similarly, the level of *R. solani* infection was the same for *OsPUB41C40A* transgenic plants irrespective of whether or not the expression of the gene is induced. In contrast, the transgenic plants carrying wild type *OsPUB41* showed a significant enhancement in tolerance to *R. solani* infection when expression of the transgene is induced (Fig. 6b). Similar results were obtained in additional transgenic lines ectopically expressing either *OsPUB41* or *OsPUB41C40A* (Additional file 11: Table S8). Apart from qualitative analysis, the relative expression level of a fungal gene [18-28S ribosomal (r)DNA] as compared to a plant gene (*AtUbq5*) between induced (Estradiol) and uninduced (DMSO) samples indicated a similar fungal load in *OsPUB41C40A* expressing lines. (Value ~ 1 indicates similar fungal load whereas a value < 1 indicates less fungal load) (Additional file 12: Fig. S4). Similar results were obtained in additional transgenic lines (Additional file 13: Table S9). This shows that unlike for wild type *OsPUB41*, the expression of *OsPUB41C40A* does not lead to enhanced tolerance to *R. solani* infection in Arabidopsis.

## Discussion

CWDEs purified from Xoo induce defense responses in rice [5]. Very little information is available about rice functions that might be involved in elaboration of CWDE-induced immune responses. In microarray analysis of rice leaves treated with either one of two different CWDEs, namely LipA or ClsA, expression of one E3 ubiquitin ligase (*OsPUB41*) was consistently induced. *OsPUB41* expression was also enhanced upon treatment with commercially available CWDEs (fungal cellulase, xylanase or pectinase). E3 ubiquitin ligases are known to involved in regulation of plant immune responses. *OsPUB41* might participate in the signaling cascade activated upon cell wall damage. Hence, an enhancement in *OsPUB41* expression is observed following cell wall damage. In addition to CWDEs, the expression of *OsPUB41* was also induced upon exposure to pathogens. *OsPUB41* expression was also induced upon exposure to several known PAMPs and DAMPs. The enhanced expression of *OsPUB41* in presence of either a CWDE or an elicitor or a pathogen hinted towards the possibility that overexpression of this gene might mimic pathogen infection and result in elicitation independent enhancement of defense responses.

Consistent with this possibility, overexpression of *OsPUB41* was found to result in induction of callose deposition in rice and Arabidopsis. Transient overexpression of *OsPUB41* imparted enhanced tolerance to Xoo infection in rice. In transgenic Arabidopsis plants ectopically expressing *OsPUB41*, enhanced tolerance to *R. solani* infection was observed. Thus, overexpression of *OsPUB41* results in enhanced defense responses in rice and Arabidopsis. It appears that this protein may have a conserved role in elaboration of innate immune responses in rice (a monocot) and in Arabidopsis (a dicot).

OsPUB41 has been earlier shown to be an E3 ligase. Mutants (OsPUB41C40A and OsPUB41V51R) of OsPUB41 that are defective in the E3 ligase activity of the protein failed to induce defense responses in rice and Arabidopsis. Hence, biochemical activity of OsPUB41 appears to be crucial for its role in induction of defense responses. The E3 ubiquitin ligases are known to play an important role in regulating plant immune signaling [9] by ubiquitination of their target proteins. The type of ubiquitination determines the fate of the substrate protein. Polyubiquitination through K48 marks the protein for degradation by 26S proteasome, whereas polyubiquitination with lysine linkages other than K48 and monoubiquitination regulate internalization and endocytotic trafficking of membrane receptors, histone modification, etc. [12]. E3 ubiquitin ligases can act as either negative or positive regulators of immune responses. For example, Rice SPL11 (Spotted Leaf11) is a negative regulator of cell death [19]. In *Arabidopsis*, the U-box E3 ligases Plant U-Box 12 (PUB12) and PUB13 attenuate PTI responses triggered upon recognition of flagellin [20]. In Chinese wild grapevine (*Vitis pseudoreticulata*), EIRP1, a RING domain E3 ligase ubiquitinates VpWRKY11 (WRKY nuclear transcription factor) [21], which is a negative regulator of immune responses, and marks it for degradation. EIRP1 overexpression in *Arabidopsis* conferred enhanced tolerance to fungal and bacterial pathogens [21]*.* In rice, the RING-type E3 ligase, XA21 binding protein 3 (XB3) interacts with the receptor kinase protein XA21, which confers resistance against Xoo [22]*.* OsPUB15 has been shown to positively regulate plant innate immunity by interacting with rice receptor-like kinase PID2 [23]. Silencing of OsPUB44 resulted in suppression of peptidoglycan and chitin-induced immune responses suggesting a positive role for OsPUB44 in rice immunity. OsPUB44 has also been reported as a target of XopP (an Xoo effector protein) which suppresses peptidoglycan mediated immune responses. The XopP protein inhibits the E3 ligase activity of OsPUB44 [24]. At the moment, it is not clear whether the induction of immune responses following overexpression of *OsPUB41* is due to the degradation of a negative regulator of innate immunity or whether it is a result of activation of a client protein through ubiquitination.

Ectopic expression of *OsPUB41* in Arabidopsis results in enhanced tolerance to *R. solani*, a necrotrophic fungal pathogen. Genes such as those encoding NADPH oxidases in Arabidopsis [25]*, OsWRKY80* [26] and chitinases [LOC_Os11g47510 [27]] in rice have been reported till date to provide enhanced tolerance to *R. solani*. Ectopic expression of *OsPUB41* in Arabidopsis leads to enhanced expression of JA biosynthetic and response genes. Studies in Arabidopsis, tomato, and rice have shown that host resistance toward necrotrophs is conferred by ethylene and JA regulated signaling networks [28–30]. Also, it was observed that resistance against necrotrophic pathogens which is triggered by β-amino-butyric acid treatment is associated with induction of callose deposition [31]. We find that ectopic expression of *OsPUB41* in Arabidopsis results in increased expression of certain JA biosynthesis and response genes and enhanced callose deposition. It is possible that the enhanced tolerance to *R. solani* in *OsPUB41* expressing Arabidopsis plants is due to induction of callose deposition and other defense responses.

*OsPUB41* overexpression leads to enhanced tolerance to Xoo infection in rice. Exogenously applied SA (and SA-mediated defenses) and JA (and JA-mediated defenses) were found to enhance tolerance to Xoo infection in rice [32–34]. In addition, overexpression of JA marker genes like *AOS2*, *MYC2* or *JAZ8* have been reported to modulate resistance to Xoo in rice [10, 34–36]. Overexpression of SA marker genes (*OsSGT*, *OsPAL*, *OsNH1* or *OsWRKY13*) is known to impart resistance to Xoo infection in rice [37–40]. Transient overexpression of *OsPUB41* in rice induces expression of markers of JA and SA signaling genes. In addition, overexpression of *OsPUB41* triggers expression of *PR* genes (*PR1a*, *PR1b*, *PR2*, *PR3*, *PR5* and *PR9*) in rice. Induction of *PR* genes is reported to be associated with enhanced tolerance to pathogen infection [41, 42]. Thus, the enhanced tolerance to Xoo infection which is observed upon *OsPUB41* overexpression might arise due to enhancement in expression of rice genes associated with JA and SA biosynthesis and response and genes from PR family. *OsPUB41* overexpression leads to enhanced tolerance to Xoo infection in rice but does not provide enhanced tolerance to Pst infection in Arabidopsis. Coronatine (a phytotoxin and a potent virulence factor of Pst) has been reported to suppress induction of callose deposition in Arabidopsis during infection [43]. Hence, although expression of *OsPUB41* induces callose deposition, it may not provide enhanced tolerance to Pst infection in Arabidopsis. Pst is a hemibiotroph which during infection activates the JA pathway in Arabidopsis [44]. SA and JA are known to be antagonistic to each other in Arabidopsis [45, 46]. Hence, by inappropriate activation of JA pathway, Pst suppresses SA pathway and SA mediated defense responses. Enhanced expression of SA responsive genes like *NPR1* and *PR* genes (*PR1*, *PR2* and *PR5*) is associated with resistance to Pst infection in Arabidopsis [47]. In addition, an alteration in expression of SA biosynthetic genes like *SID2* and *PAL* (*PAL1-PAL4* genes) affect disease (caused by Pst) outcome in Arabidopsis [48, 49]. *OsPUB41* expression in Arabidopsis induces expression of JA marker genes but has no effect on expression of SA marker genes (biosynthetic as well as response genes). It is possible that since the expression of SA related genes (biosynthetic and response genes) remained unaffected in Arabidopsis upon *OsPUB41* expression, neither resistance nor enhanced susceptibility to Pst infection was observed. Therefore, it is possible, that although both Xoo and Pst are hemibiotrophic in nature, *OsPUB41* expression provides enhanced tolerance to Xoo in rice but not to Pst in Arabidopsis.

There are certain differences in hormonal pathways in rice and Arabidopsis. For example: AtPAD4 (Phytoalexin deficient 4) is SA responsive and has a positive feedback/regulation with respect to SA. However, its orthologue, OsPAD4 interacts with both JA and SA signaling in rice [50]. In addition, role of AtEDS1 (Enhanced disease susceptibility) in plant (Arabidopsis) defense depends on SA signaling. However, role of OsEDS1, rice orthologue of AtEDS1, in plant (rice) defense depends on JA signaling. Expression of AtEDS1 in oseds1 (OsEDS1 knockout rice plants) partially complements oseds1 defense phenotype [51]. Apart from known commonalities, there exist quite a few subtle differences in the regulation and execution of defense responses in rice and Arabidopsis (monocots and dicots). Hence, it is possible that *OsPUB41* induces expression of genes differently in rice and in Arabidopsis.

In summary, *OsPUB41* expression is induced following treatment of rice leaves with various CWDEs. Also, overexpression of *OsPUB41* leads to induction of plant defense responses. This suggests that *OsPUB41* might be involved in elaboration of CWDE-induced plant immune responses. At this point, we do not know whether *OsPUB41* is essential for elicitation of rice immune responses following treatment with CWDEs. To address this issue, *OsPUB41* knockdown or knockout rice plants need to be generated using either RNAi or genome editing and their phenotypes need to be assessed following treatment with CWDEs.

## Conclusion

*OsPUB41* might be a positive regulator of innate immunity that participates in PAMP and DAMP triggered signaling pathways.

## Methods

### Plant materials and growth conditions

Ten-fifteen-days-old seedlings of greenhouse grown bacterial blight susceptible rice cultivar, Taichung Native-1 (TN-1) [procured from Indian Institute of Rice Research (IIRR) and IIRR procured TN-1 from International Rice Research Institute (original source)], were used for qPCR and callose deposition. Forty-days-old, greenhouse-grown TN-1 plants were used for Xoo infection assays. The *Arabidopsis thaliana* (Arabidopsis) Columbia ecotype (Col-0) was used as wild type and for generating transgenic plants [Col-0 seeds from laboratory stock were used by the authors for all experiments. Seeds for maintaining laboratory stock were initially procured from Dr. Imran Siddiqi’s Lab (CCMB, India) who had originally procured them from TAIR (original source)]. Arabidopsis plants were grown and maintained as described earlier [18].

### Generation of plant expression plasmids (pMDC7-OsPUB41, pMDC7-OsPUB41C40A and pMDC7-OsPUB41V51R) using gateway cloning

The *OsPUB41* gene (LOC_Os03g13740) encodes a CDS of length 1338 bps. The cDNA was prepared using Superscript III reverse transcriptase (Invitrogen) from total RNA isolated from LipA treated TN-1 rice leaves (2 h post-treatment) as described previously [8]. The *OsPUB41* gene was cloned into the inducible plant expression vector, pMDC7, by Gateway cloning as per the manufacturer’s instructions (Invitrogen). Entry and destination constructs (pENTR-OsPUB41 and pMDC7-OsPUB41, respectively) were generated in this process. Entry constructs for two OsPUB41 mutants (pENTR-OsPUB41C40A and pENTR-OsPUB41V51R) containing substitutions (corresponding to amino acid residues) either at 40th (C to A) or 51st (V to R) position were constructed using Quick change site-directed mutagenesis kit (Stratagene), primers with altered codons (Additional file 18: Table S11) and pENTR-OsPUB41 (as a template). From entry constructs of OsPUB41 mutants, destination constructs: pMDC7-OsPUB41C40A and pMDC7-OsPUB41V51R were obtained. In pMDC7, gene expression is under the control of the 17-β-estradiol inducible XVE promoter. These plant expression vectors containing either *OsPUB41* or its mutant forms were transformed into *Agrobacterium tumefaciens* LBA4404 strain by electroporation and selected on medium containing appropriate antibiotics (Additional file 19: Table S12). The LBA4404/pMDC7-OsPUB41, LBA4404/pMDC7-OsPUB41C40A and LBA4404/pMDC7-OsPUB41V51R clones were confirmed by colony PCR and subsequent sequencing of the PCR amplicons using vector specific primers (Additional file 18: Table S11).

### Generation of clones for bacterial expression of *OsPUB41* and its mutant forms (*OsPUB41C40A* and *OsPUB41V51R*)

*OsPUB41*, *OsPUB41C40A* and *OsPUB41V51R* were amplified using pENTR-OsPUB41, pENTR-OsPUB41C40A and pENTR-OsPUB41V51R as templates respectively with KpnIF and Kpn1RNS primers (Additional file 18: Table S11). Modified pETM40 (MpETM40) vector and the amplicons were digested with KpnI. The digested vector was treated with Antarctic phosphatase and ligated with either of the aforementioned KpnI digested amplicons. The ligation mixture was used for transforming *E. coli* DH5α cells. Clones were selected using appropriate antibiotics (Additional file 19: Table S12) and subsequently confirmed by PCR and sequencing using MBPF and KpnIRNS primers (Additional file 18: Table S11). MpETM40-OsPUB41, MpETM40-OsPUB41C40A and MpETM40-OsPUB41V51R were subsequently transformed into *E. coli* BL21-DE3 individually for expression of the respective proteins. The *E. coli* BL21-DE3 clones were further selected using appropriate antibiotics (Additional file 19: Table S12) and subsequently confirmed by PCR and sequencing using MBPF and KpnIRNS primers (Additional file 18: Table S11).

### Generation of transgenic Arabidopsis plants

Arabidopsis Col-0 (wild type) was used to generate transgenic lines expressing either *OsPUB41* or *OsPUB41C40A* (mutant form of *OsPUB41*) by floral dip method of transformation [52] using *Agrobacterium* strain LBA4404 with either pMDC7-OsPUB41 or pMDC7-OsPUB41C40A construct. During germination, the transformants were selected using hygromycin (25μgml^− 1^). Putative transgenic plants (hygromycin resistant T_1_ generation) were further confirmed by direct PCR (Terra PCR direct polymerase kit, Clontech) using leaf tissue and sequencing of the amplified product. Plants from three independent lines expressing either *OsPUB41* or *OsPUB41C40A* (genotype confirmed, T_2_ generation), were used in all experiments.

### Analyses of publicly available microarray data from GEO database

The dotCEL files (.CEL) for LipA (GEO-ID: GSE53940 and GSE49242), ClsA (GEO-ID: GSE8216), *Magnaporthe grisea* FR13 (GEO-ID: GSE7256), *Magnaporthe oryzae* Guy11 (GEO-ID: GSE18361), *PXO99A* and *PXO86* (GEO-ID: GSE36272) were downloaded from GEO (https://www.ncbi.nlm.nih.gov/geo/). PLIER normalized dotCHP (.CHP) files were generated for these dotCEL files using Affymetrix Expression Console software (ThermoFisher Scientific). Using the Affymetrix Transcriptome Analysis Console these PLIER normalized dotCHP files were analysed to obtain relative fold change values for treated versus mock. Only relative fold change values for *OsPUB41* with *p* value < 0.05 have been tabulated.

### Treatment of Rice leaves with CWDEs, elicitors or Xoo pathogen

Ten-fifteen-days-old, TN-1 plants were infiltrated with one of the following:

Xylanase, Pectinase, Cellulase (Sigma, 2.35 units each, mock: water), Sucrose (Sigma, 1 mM, mock: water), ATP (Calbiochem, 1 mM, mock: water), Flg22 (Genscript, 1 mM, mock: water) lipopolysaccharide [LPS purified from Xoo, 100 μg ml^− 1^; method for LPS purification is as described [53], mock: water] and Xoo (BXO43 strain, resuspended in water, mock: water). Twelve hours post treatment, rice leaves were harvested for q-RTPCR assays.

### Callose deposition assay

Callose deposition in rice was assayed upon transient overexpression of *OsPUB41* or mutated forms of *OsPUB41* (C40A and V51R). *Agrobacterium* strains containing either pMDC7-OsPUB41, pMDC7-OsPUB41C40A or pMDC7-OsPUB41V51R were grown, induced (by Acetosyringone) and infiltrated into rice leaves, either with the inducer (Estradiol) or without the inducer (DMSO) as previously described [18]. Twelve hours post infiltration these rice leaves were cut, processed, stained (with aniline blue) and viewed under an epifluorescence microscope, as previously described [18].

Callose deposition assays were also performed in stable transgenic Arabidopsis lines expressing either *OsPUB41* or *OsPUB41C40A* under a 17–β–estradiol inducible system. Either inducer (Estradiol) or mock (DMSO) solution was infiltrated in the rosette stage leaves of T_2_ generation of *OsPUB41* or *OsPUB41C40A* expressing plants using a needleless 1 ml syringe. Twelve hours post-infiltration, leaves were collected, processed and the assay for visualization of callose deposition was performed as mentioned above for rice leaves.

### Xoo infection assay in rice

*OsPUB41* or its mutant forms were transiently overexpressed in midveins of the leaves of 40-days-old rice plants using either LBA4404/pMDC7-OsPUB41 or LBA4404/pMDC7-OsPUB41C40A or LBA4404/pMDC7-OsPUB41V51R cultures as described earlier [18]. Twelve hours later, the midveins of the leaves were infected with wild type Xoo (BXO43 strain) by pricking with a needle touched to a pellet of saturated culture. Lesion lengths were measured on the twelfth day post-infection.

### Western-blotting

Leaves of fourteen-days-old TN-1 rice seedlings were infiltrated with *Agrobacterium* strains containing either pMDC7-OsPUB41, pMDC7-OsPUB41C40A or pMDC7-OsPUB41V51R constructs with or without estradiol. Infiltrated leaf samples were collected after 12 h and ground in liquid nitrogen followed by homogenization in lysis buffer (50 mM Tris-HCl, pH 7.5, 150 mM NaCl, 250 mM Mannitol, 5 mM EDTA, 10% glycerol, 1 mM DTT, 1% Triton X-100, 1 mM PMSF and 1 mM NaF) [54]. After centrifugation (15,000 g, 15 min, 4 °C), equal amounts of isolated protein supernatants were separated using two 15% SDS-PAGE gels. Each sample was split into two: first half was loaded in Gel 1 and second half of the same sample was loaded in Gel2. The OsPUB41 protein and its mutant forms were detected by Western blot analysis (Gel1 was used for this purpose) using polyclonal rabbit anti-OsPUB41Pep3 antibody [1: 1000 dilution, Genscript generated Ab using a peptide (Pep3; sequence: VAESAARRGAAGRAC) of OsPUB41]. Rabbit polyclonal Histone (H3, Abcam) antibody (1: 50,000) was used to detect Histone (loading control) in these samples (Gel2 was used for this purpose). HRP conjugated anti-rabbit secondary antibody (Abcam) was used and the protein bands were viewed using Luminata Forte HRP substrate (Millipore). Chemiluminescence imaging system with Chemi-capt 5000 software (version 12.8; Vilber Lourmat) was used for capturing the signal. Induced and uninduced (for *OsPUB41* expression) leaf tissues of Arabidopsis were processed in a similar manner.

For purified proteins (OsPUB41, OsPUB41C40A, OsPUB41V51R and MBP), immunoblotting was performed using mouse monoclonal anti-polyhistidine alkaline phosphatase antibody (1: 4000 dilution, Sigma). Approximately 100 ul of chromogenic substrate (Sigma, 66 μl NBT stock + 33 μl BCIP) in 10 ml of alkaline phosphatase buffer (100 mM NaCl, 5 mM MgCl_2_ and 100 mM Tris-Cl, pH 9.5) was added to the blot and incubated with gentle shaking. The bands appeared in 1–2 min and the reaction was stopped by washing the blot with water.

### *Rhizoctonia solani* AG1-IA infection in Arabidopsis seedlings

Arabidopsis seedlings (Col-0, *OsPUB41* and *OsPUB41C40A*) were grown for 15 days on vertically positioned agar plates containing ½ MS with inducer (Estradiol) or without the inducer (DMSO). *R. solani* infection was carried out as described previously [55]. Seven dpi, seedlings were washed twice with sterile water to remove superficially growing fungus and stained, for 1 min, with Trypan Blue solution (10 ml Lactic acid, 10 ml glycerol, 10 ml water, 10 g phenol, 10 g Trypan Blue) diluted in 96% ethanol in 1: 2 ratio. After three washes with destaining solution (10 ml Lactic acid, 10 ml glycerol, 10 ml MQ, 10 g phenol, diluted in 96% ethanol in 1: 2 ratio), seedlings were observed under the light microscope (10X objective).

### Quantification of *R. solani* DNA from infected Arabidopsis leaves by real-time PCR

For fungal load determination, DNA was isolated from Arabidopsis seedlings (Col-0, *OsPUB41* and *OsPUB41C40A*), 7 dpi using the CTAB method [56]. Two ng of total DNA from each infected plant tissue sample was used for qPCR. UBQ5F and UBQ5R (plant specific) and Rs1F and Rs2R (fungus specific) primers (Additional file 18: Table S11) [57] were used for qPCR performed on the Applied Biosystems ViiA7 Real-Time PCR System, using PowerSYBR Green/ROX Master Mix (ThermoFisher Scientific). The relative expression level of the fungal gene as compared to the plant gene between induced (Estradiol) and uninduced (DMSO) samples was calculated using the 2^(−ΔΔCt)^ method [58].

### Quantitative real time PCR (qRT-PCR)

Total RNA from Arabidopsis and rice leaves was isolated using TRIzol Reagent (ThermoFisher Scientific). After DNaseI treatment (NEB, according to manufacturer’s instructions), cDNA was synthesized with 1 μg of total RNA by Oligo (dT)-primed reverse transcription using EcoDry kit (Clontech, Takara, USA). qRT-PCR was performed on the Applied Biosystems ViiA 7 Real-Time PCR System using PowerSYBR Green/ROX Master Mix (ThermoFisher Scientific). *OsActin* and *AtUbq5* were used as internal controls for rice and Arabidopsis respectively. The primers used for qPCR have been listed in (Additional file 18: Table S11). The relative expression of various genes between induced (Estradiol) and uninduced (DMSO) samples was calculated using the 2^(−ΔΔCt)^ method [58].

### *Pseudomonas syringae* pv. *tomato* DC3000 (Pst) infection assay in Arabidopsis plants

Cultures of virulent Pst were grown to mid to late log phase in LB supplemented with rifampicin (50 μg ml^− 1^) at 28 °C for 12 h before inoculation. Fully expanded leaves of wild-type and transgenic Arabidopsis plants were pressure infiltrated with either Pst (OD_600_ of 0.02) suspended in 10 mM MgCl_2_ with estradiol (inducer) or DMSO (control; uninduced) using a needleless syringe into three leaves per plant [59]. In each experiment, three plants were used per time point per condition (induced or uninduced) for each transgenic line. Bacterial growth assays were performed at 0 and 48 h post infection (hpi) to determine disease progression in the plants. Leaves were surface sterilised with 70% (v/v) ethanol and then washed in sterile water for 1 min. Each leaf was placed in 500 μl of 10 mM MgCl_2_ solution and crushed to acquire the bacteria. The resulting solution was serial diluted and 10 μl of each dilution was spotted on LB plates containing 50 μg ml^− 1^ rifampicin. The plates were incubated at 28 °C for 48 h prior to counting of the colonies.

### Overexpression and purification of OsPUB41 and its mutant forms

Single colonies of selected BL21-DE3 clones containing either MpETM40-OsPUB41 or MpETM40-OsPUB41C40A or MpETM40-OsPUB41V51R or MpetM40 (empty vector), were inoculated into 5 ml LB medium containing 100 μg ml^− 1^ Kanamycin and grown overnight (37 °C, 200 rpm). Further, 300 ml LB medium containing 100 μg ml^− 1^ Kanamycin was inoculated with 1% inoculum of the overnight grown culture. The cultures were grown until mid-log phase (OD_600_ ~ 0.4–0.6). After addition of IPTG (inducer: 1 mM), the cultures were further incubated (37 °C, 200 rpm) for 4–6 h. At the same time, uninduced cultures were also maintained. One ml of each culture was pelleted and used for SDS-PAGE analysis. The cultures were centrifuged (8000 rpm, 4 °C, 10 min). The cell pellet from the remaining 299 ml culture of *E. coli* expressing 6X-His-tagged recombinant protein was resuspended in 10 ml of Resuspension Buffer (50 mM Tris-HCl (pH 8), 150 mM NaCl) containing 1 mM PMSF. The cells were disrupted by sonication on ice and centrifuged (12,000 g, 30 min, 4 °C). The supernatants were added to TALON beads pre-equilibrated with Resuspension Buffer. It was then kept overnight at 4 °C on a low speed rocker. Columns with these TALON beads were prepared in 50 ml falcons. The column was washed thrice with 50 ml of Wash Buffer-1 (50 mM Tris-HCl (pH 8), 150 mM NaCl, 10 mM Imidazole). After 3 washes with Wash Buffer-2 (50 mM Tris-HCl (pH 8), 500 mM NaCl), proteins were eluted using Elution Buffer (50 mM Tris-HCl (pH 8), 150 mM NaCl, 100 mM Imidazole). The purified proteins were aliquoted and stored at − 80 °C until further use. OsPUB41 protein and its mutant forms have an N-terminal MBP tag and a C-terminal 6X-His tag. The empty vector (MpETM40), expresses MBP protein with a C-terminal 6X-His tag.

### In vitro self-ubiquitination assay

Ubiquitination reactions of 100ul each containing either of the purified *E. coli* expressed proteins; OsPUB41, OsPUB41C40A, OsPUB41V51R and MBP (10 uM each) along with ubiquitination reaction buffer (50 mM Tris-HCl, pH 7.5, 5 mM MgCl_2_, 1 mM dithiothreitol (DTT), 2 mM ATP), human E1, E2 UbcH5A (R&D Biosciences) and His-tagged penta-ubiquitin were incubated for 4 h at 37 °C. The reaction was stopped by the addition of SDS sample buffer. After boiling for 10 min, the samples were separated by 15% SDS-PAGE and subjected to immunoblotting using either of the following antibodies: polyclonal rabbit anti-MBP antibody (1: 2000 dilution, Abcam) or monoclonal mouse anti-polyHis antibody conjugated to alkaline phosphatase (1: 4000 dilution, Sigma) or mouse monoclonal anti-ubiquitin antibody (1: 1000 dilution, Santa Cruz Biotechnology). Donkey polyclonal antibody to rabbit IgG (1: 50,000 dilution, Abcam) or goat polyclonal antibody to mouse IgG (1: 2000 dilution, Abcam), conjugated to HRP was used as secondary antibody (1 h, at room temperature). Anti-His probed blot was developed as described above using a chromogenic substrate (NBT and BCIP).

### Statistics

Unless mentioned, all experiments were performed atleast in three biological replicates. All experiments in Arabidopsis were reproduced in three independent transgenic lines. Statistical analysis was performed using the unpaired students t–test for independent means wherever necessary. One-way ANOVA followed by the Tukey-Kramer honestly significant difference test was used for analysing qPCR and scoring data for *R. solani* infection in Arabidopsis.

## Supplementary information

**Additional file 1: Table S1.** Expression of *OsPUB41* is induced following treatment of rice leaves with either DAMPs or PAMPs.

**Additional file 2: Table S2.** *OsPUB41* expression is induced following infection with either bacterial or fungal pathogens.

**Additional file 3: Fig. S1.** Domain organization of OsPUB41 and Multiple Sequence Alignment with other plant E3 ubiquitin ligases depicting the conserved residues. InterPro [14] analysis revealed that OsPUB41 protein has an N-terminal U-box domain and an armadillo type fold (A). BLAST search for OsPUB41 (LOC_Os03g13740) revealed a list of homologous proteins from various plant species. Ten different plant U-box domain containing proteins (PUBs) from different species from this list (first ten) were chosen for multiple sequence alignment (MSA). T-coffee tool from NCBI was used for generating the MSA (B). Homologous sequences with at least 40% identity were used for MSA. MSA file was edited using ESPript tool [60]. Red and yellow colours represent invariant and conserved amino acids respectively. Black boxes represent SDM mutations: C40A and V51R.

**Additional file 4: Fig. S2.** Transient overexpression of *OsPUB41* and its mutant forms (*OsPUB41C40A* and *OsPUB41V51R*) in rice leaves (Confirmation by qPCR and Western blotting). Leaves (*n* = 20) of 10–15 days old TN-1 rice plants were infiltrated with suspension of *Agrobacterium* strain (100 μl per leaf), containing either pMDC7-OsPUB41, pMDC7-OsPUB41C40A or pMDC7-OsPUB41V51R, with the inducer (40 μM 17-β-estradiol dissolved in 0.1% DMSO) or without the inducer (0.1% DMSO) using needleless 1 ml syringes. After 12 h, leaves were collected, crushed and processed for either western blotting or qPCR. *OsActin* was used as internal control for qPCR. The graph represents relative fold change (2^-∆∆Ct^) using expression values of induced over uninduced samples (A). Student’s two-tailed t-test for independent means was performed on delta C_t_ values to test for significance (*p* < 0.05). Each sample was split into two: first half was loaded in Gel 1 and remaining half of the same sample was loaded in Gel2. The OsPUB41 protein and its mutant forms were detected (Gel1) using anti-OsPUB41Pep3 antibody (approximate size is 47 kDa). Rabbit polyclonal Histone (H3, Abcam) antibody (1: 50,000) was used to detect Histone (Gel2: loading control, approximate size: 17 kDa; represented by lower panel of Fig. S2B) in these samples. Leaf sample of transgenic Arabidopsis expressing *OsPUB41* was used as a positive control (B).

**Additional file 5: Table S3.** Estradiol (by itself / alone) does not induce callose deposition in rice or Arabidopsis.

**Additional file 6: Table S4.** Estradiol (by itself / alone) does not affect Xoo infection in rice.

**Additional file 7: Table S5.** Estradiol (by itself / alone) does not affect expression of defense genes in rice and Arabidopsis.

**Additional file 8: Fig. S3.** Estradiol inducible (ectopic) expression of *OsPUB41* and *OsPUB41C40A* in transgenic Arabidopsis plants. Leaves of three weeks old plants were infiltrated either with inducer (40 μM 17-β-estradiol) or with DMSO using a 1 ml needleless syringe. Twelve hours post infiltration, leaves were harvested and processed for qPCR analysis. The graph represents relative fold change (2^-∆∆Ct^) using expression values of induced over uninduced samples. *AtUbq5* was used as an internal control for qPCR analysis. Three biological repeats were performed for each independent transgenic line. Similar results were obtained in three independent transgenic lines. Student’s two-tailed t-test for independent means was performed on delta C_t_ values to test for significance (*p* < 0.05).

**Additional file 9: Table S6.** Callose deposition assay: Data from three transgenic Arabidopsis lines ectopically expressing either *OsPUB41* or *OsPUB41C40A.*

**Additional file 10: Table S7.** Ectopic expression of *OsPUB41* leads to enhanced expression of Arabidopsis genes involved in JA biosynthesis and response, but does not induce SA biosynthetic and response genes: data from three transgenic Arabidopsis lines.

**Additional file 11: Table S8.** *Rhizoctonia solani* AG1-1A infection assay in Arabidopsis: Data from three transgenic Arabidopsis lines ectopically expressing either *OsPUB41* or *OsPUB41C40A.*

**Additional file 12: Fig. S4.** Determination of fungal (*Rhizoctonia solani* AG1-1A) load during infection in Arabidopsis seedlings ectopically expressing either *OsPUB41* or *OsPUB41C40A.* For quantitative assessment of fungal load, DNA was isolated from infected Arabidopsis seedlings (Col 0, *OsPUB41* and *OsPUB41C40A*, 7 dpi) and used for qPCR. UBQ5F and UBQ5R (plant specific for *AtUbq5* gene) and Rs1F and Rs2R (fungus specific for ITS region of 18-28S rDNA) primers (Additional file 18: Table S11) were used for qPCR. Graph represents relative level of amplification of fungal gene as compared to plant gene between induced (with Estradiol) and uninduced (with 0.1% DMSO) samples. This was calculated using the 2^(−ΔΔCt)^ method. Three biological repeats were performed for each sample using 3 independent lines. One-way ANOVA was used to test for significance, followed by Tukey-Kramer honestly significance difference test (*p* < 0.05, represented by letters ‘a’ and ‘b’).

**Additional file 13: Table. S9.** Determination of fungal (*Rhizoctonia solani* AG1-1A) load during infection in Arabidopsis seedlings ectopically expressing either *OsPUB41* or *OsPUB41C40A*: data from three transgenic Arabidopsis lines

**Additional file 14: Fig. S5.** Pst infection assay in transgenic Arabidopsis plants ectopically expressing *OsPUB41*. The graph represents average number of colony forming units of Pst per leaf at 0 and 48 h post infection from Col 0 and transgenic Arabidopsis plants. Y axis is logarithmic (log scale). Error bars represent standard error. Student’s two-tailed t-test for independent means was performed to test for significance (p < 0.05). Similar results were obtained in three independent experiments and in three independent transgenic lines.

**Additional file 15: Table S10.** Pst infection assay in Arabidopsis: Data from three transgenic Arabidopsis lines ectopically expressing *OsPUB41*

**Additional file 16: Fig. S6.** The OsPUB41, OsPUB41C40A and OsPUB41V51R proteins were purified from *E. coli.* Bacterially expressed 6X-His-tagged MBP, OsPUB41, OsPUB41C40A and OsPUB41V51R proteins were purified, separated by 10% SDS-PAGE and further subjected to immunoblot analysis with anti-His antibody. Lanes 1, 2, 3 and 4 represent MBP (~ 43 kDa), OsPUB41, OsPUB41C40A and OsPUB41V51R (~ 90 kDa each) respectively.

**Additional file 17: Fig. S7.** The C40A and V51R mutations affect E3 ubiquitin ligase activity of OsPUB41. OsPUB41 protein has been shown to be a biochemically active, polyubiquitinating E3 ubiquitin ligase, by an in vitro auto-ubiquitination assay [13]. In order to generate biochemically inactive versions of OsPUB41, two independent mutants (OsPUB41C40A and OsPUB41V51R) in the U-box domain were generated. These residues (Cysteine at 40th position and Valine at 51st position) were selected because they were highly conserved across various homologues of OsPUB41 (Fig. S1B, Multiple Sequence Alignment for OsPUB41). Also it has been shown that mutation of the corresponding residues in the U-box E3 ubiquitin ligases, rice SPL11 (Valine to Arginine) and tobacco NtCMPG1 (Cysteine to Alanine), respectively, led to abolition of E3 ligase activity [19, 61]. OsPUB41 protein and its mutant forms were expressed and purified from *E.coli*, and tested for their E3 ligase activity using an in vitro auto-ubiquitination assay. Purified 6X-His-tagged OsPUB41 or OsPUB41C40A or OsPUB41V51R or MBP protein was incubated with ATP, 6X-His-tagged PentaUb (Ubiquitin), E1 (Ubiquitin activating enzyme; human E1) and E2 (Ubiquitin conjugating enzyme; UbcH5A) at 37 °C for four hours. The minus and the plus symbols represent absence or presence, respectively, of indicated component of the reaction mixture (A). The reaction mixtures were then resolved by 15% SDS-PAGE and subjected to immunoblot analysis with either anti-His antibody (B) or anti-Ubiquitin antibody (C) or MBP antibody (D). OsPUB41 (lane 6) protein was found to undergo polyubiquitination (bands corresponding to ≥90 kDa) whereas its mutant forms; OsPUB41C40A and OsPUB41V51R failed to exhibit E3 ligase activity (lanes 7 and 8). MBP does not affect the ubiquitination reaction (lane 9: Tag control). Lanes 3 and 4 are flipped in anti-His and anti-Ubiquitin blots as reaction mixtures loaded in these lanes (contain indicated reaction components) are according to numbers “3” and “4” as mentioned in the Table (A).

**Additional file 18: Table S11.** List of primers.

**Additional file 19: Table S12.** List of strains, plasmids and antibiotics [62–64].

## Data Availability

All data generated or analyzed during this study are included in this published article (and its additional files). Any material generated during the current study is available from the corresponding author on reasonable request.

## Abbreviations

CbsA: cellobiosidase
ClsA: Cellulase A
CWDE: cell wall degrading enzyme
DAMP: Damage associated molecular patterns
DMSO: dimethyl sulfoxide
dpi: days post infection or infiltration
Est: 17-β-Estradiol
hr.: hour
JA: Jasmonic acid
JA-Ile: (+)-7-iso-Jasmonoyl-L-isoleucine
LipA: Lipase/esterase A
LPS: lipopolysaccharide
MBP: Maltose binding protein
MS: Murashige and Skoog’s
MSA: multiple sequence alignment
PAMPs: pathogen-associated molecular patterns
*PR*: *Pathogenesis Related*
Pst: *Pseudomonas syringae* pv. *tomato* DC3000
PTI: PAMP-triggered immunity
PUB: Plant U-Box
*R. solani*: *Rhizoctonia solani* AGI-1A
ROS: Reactive oxygen species
SA: Salicylic acid
TN-1: Taichung Native-1
Xoo: *Xanthomonas oryzae* pv. *oryzae*
XynB: xylanase
