# Supplementary material for: Overexpression of OsPUB41, a Rice E3 ubiquitin ligase induced by cell wall degrading enzymes, enhances immune responses in Rice and Arabidopsis
Source: BMC Plant Biol. 2019 Nov 29;19:530. doi: 10.1186/s12870-019-2079-1 (PMC6884774; doi:10.1186/s12870-019-2079-1)
Supplement: Supplementary file 1 — Additional file 1: Table S1. Expression of OsPUB41 is induced following treatment of rice leaves with either DAMPs or PAMPs. [file 12870_2019_2079_MOESM1_ESM.docx]

**Table S1. Expression of *OsPUB41* is induced following treatment of rice leaves with either DAMPs or PAMPs**

| **Mean fold change in *OsPUB41* expression 12h post treatment (qPCR data^a^)** | | | |
| --- | --- | --- | --- |
| **DAMPs** | | **PAMPs** | |
| **eATP** | **Sucrose** | **Flg22** | **LPS** |
| 5.2 ± 0.4 | 4.2 ± 0.7 | 7.7 ± 0.1 | 2.3 ± 0.4 |

^a^Relative fold change of *OsPUB41* (average value from three independent experiments, ± represents standard error) when leaves of ten-fifteen days old seedlings of Taichung Native-1 (TN-1) rice variety were pressure infiltrated using a needleless syringe with either DAMPs (1mM eATP or 1mM Sucrose) or PAMPs (1mM Flg22 or 100ug ml^-1^ LPS) as compared to mock (water) treatment. *OsActin* was used as an internal control in qPCR for rice. Three biological repeats were performed for each treatment. Student’s two-tailed t-test for independent means was performed on delta C_t_ values to test for significance (p < 0.05).
