# Supplementary material for: Overexpression of OsPUB41, a Rice E3 ubiquitin ligase induced by cell wall degrading enzymes, enhances immune responses in Rice and Arabidopsis
Source: BMC Plant Biol. 2019 Nov 29;19:530. doi: 10.1186/s12870-019-2079-1 (PMC6884774; doi:10.1186/s12870-019-2079-1)
Supplement: Supplementary file 2 — Additional file 2: Table S2. OsPUB41 expression is induced following infection with either bacterial or fungal pathogens. [file 12870_2019_2079_MOESM2_ESM.docx]

**Table S2. *OsPUB41* expression is induced following infection with either bacterial or fungal pathogens**

| **Experiment** | **Fungal strains** | | | | | **Bacterial strains** | | |
| --- | --- | --- | --- | --- | --- | --- | --- | --- |
|  | ***Magnaporthe grisea* FR13**^a^ | | ***Magnaporthe oryzae***  **Guy11**^a^ | | | ***PXO99A***^a^ | ***PXO86***^a^ | ***BXO43^b^*** |
| Time points^c^ | 3dpi | 4dpi | 2dpi | 4dpi | 6dpi | 24hpi | 24hpi | 12hpi |
| *OsPUB41* (Fold Change)^d^ | 7.2 | 199 | 151.7 | 19.4 | 24.2 | 7.2 | 21.3 | 5.1±0.6 |

^a^Data in publicly available databases was analyzed. In these experiments, rice had been infected with either fungal (*Magnaporthe grisea* FR13; Rice cultivar: Nipponbare; GEO-ID: GSE7256 or *Magnaporthe oryzae* Guy11; Rice cultivar: Nipponbare; GEO-ID: GSE18361) or bacterial pathogens (Xoo strains: *PXO99A* and *PXO86*, rice cultivar: IR24; GEO-ID: GSE36272)

^b^BXO43: Xoo (BXO43 strain) treated 15 days old TN-1 rice leaves were harvested 12 hpi for qPCR analysis. Relative expression of *OsPUB41* in infected as compared to mock inoculated rice leaves was calculated using the 2^(-ΔΔCt)^ method.

^c^dpi: days post infection, hpi: hours post infection

^d^Relative expression of *OsPUB41* in infected as compared to mock inoculated rice plants was calculated using Expression Console and Transcriptome Analysis Console (p ≤ 0.05).
