# Supplementary figures and images for: Overexpression of OsPUB41, a Rice E3 ubiquitin ligase induced by cell wall degrading enzymes, enhances immune responses in Rice and Arabidopsis
Source: BMC Plant Biol. 2019 Nov 29;19:530. doi: 10.1186/s12870-019-2079-1 (PMC6884774; doi:10.1186/s12870-019-2079-1)

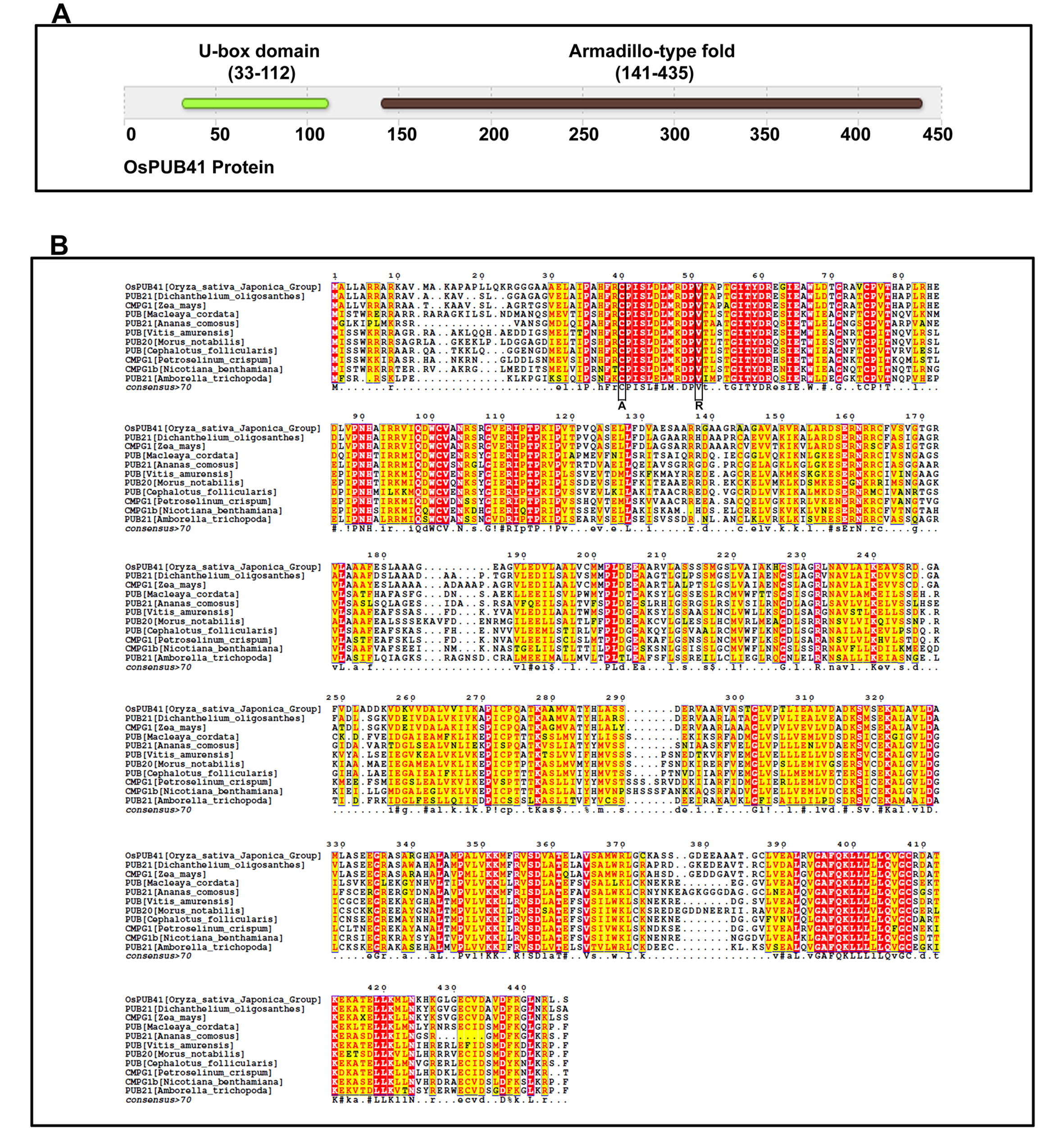

Supplement: Supplementary file 3 — Additional file 3: Fig. S1. Domain organization of OsPUB41 and Multiple Sequence Alignment with other plant E3 ubiquitin ligases depicting the conserved residues. InterPro [14] analysis revealed that OsPUB41 protein has an N-terminal U-box domain and an armadillo type fold (A). BLAST search for OsPUB41 (LOC_Os03g13740) revealed a list of homologous proteins from various plant species. Ten different plant U-box domain containing proteins (PUBs) from different species from this list (first ten) were chosen for multiple sequence alignment (MSA). T-coffee tool from NCBI was used for generating the MSA (B). Homologous sequences with at least 40% identity were used for MSA. MSA file was edited using ESPript tool [60]. Red and yellow colours represent invariant and conserved amino acids respectively. Black boxes represent SDM mutations: C40A and V51R. [file 12870_2019_2079_MOESM3_ESM.tif]

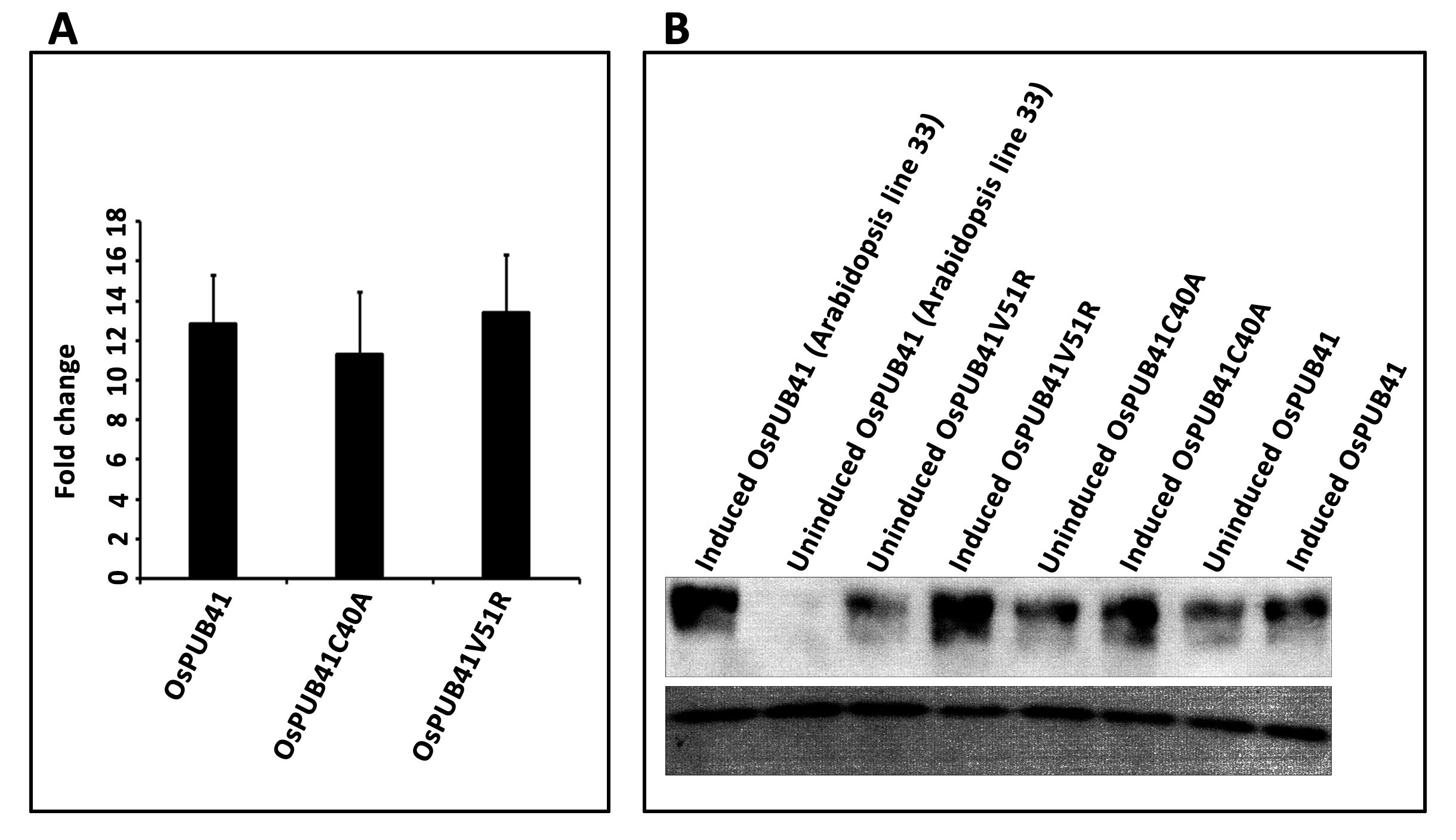

Supplement: Supplementary file 4 — Additional file 4: Fig. S2. Transient overexpression of OsPUB41 and its mutant forms (OsPUB41C40A and OsPUB41V51R) in rice leaves (Confirmation by qPCR and Western blotting). Leaves (n = 20) of 10–15 days old TN-1 rice plants were infiltrated with suspension of Agrobacterium strain (100 μl per leaf), containing either pMDC7-OsPUB41, pMDC7-OsPUB41C40A or pMDC7-OsPUB41V51R, with the inducer (40 μM 17-β-estradiol dissolved in 0.1% DMSO) or without the inducer (0.1% DMSO) using needleless 1 ml syringes. After 12 h, leaves were collected, crushed and processed for either western blotting or qPCR. OsActin was used as internal control for qPCR. The graph represents relative fold change (2-∆∆Ct) using expression values of induced over uninduced samples (A). Student’s two-tailed t-test for independent means was performed on delta Ct values to test for significance (p < 0.05). Each sample was split into two: first half was loaded in Gel 1 and remaining half of the same sample was loaded in Gel2. The OsPUB41 protein and its mutant forms were detected (Gel1) using anti-OsPUB41Pep3 antibody (approximate size is 47 kDa). Rabbit polyclonal Histone (H3, Abcam) antibody (1: 50,000) was used to detect Histone (Gel2: loading control, approximate size: 17 kDa; represented by lower panel of Fig. S2B) in these samples. Leaf sample of transgenic Arabidopsis expressing OsPUB41 was used as a positive control (B). [file 12870_2019_2079_MOESM4_ESM.tif]

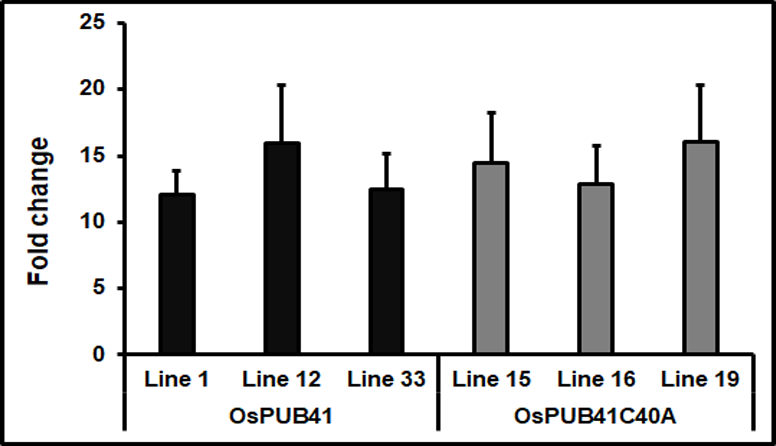

Supplement: Supplementary file 8 — Additional file 8: Fig. S3. Estradiol inducible (ectopic) expression of OsPUB41 and OsPUB41C40A in transgenic Arabidopsis plants. Leaves of three weeks old plants were infiltrated either with inducer (40 μM 17-β-estradiol) or with DMSO using a 1 ml needleless syringe. Twelve hours post infiltration, leaves were harvested and processed for qPCR analysis. The graph represents relative fold change (2-∆∆Ct) using expression values of induced over uninduced samples. AtUbq5 was used as an internal control for qPCR analysis. Three biological repeats were performed for each independent transgenic line. Similar results were obtained in three independent transgenic lines. Student’s two-tailed t-test for independent means was performed on delta Ct values to test for significance (p < 0.05). [file 12870_2019_2079_MOESM8_ESM.tif]

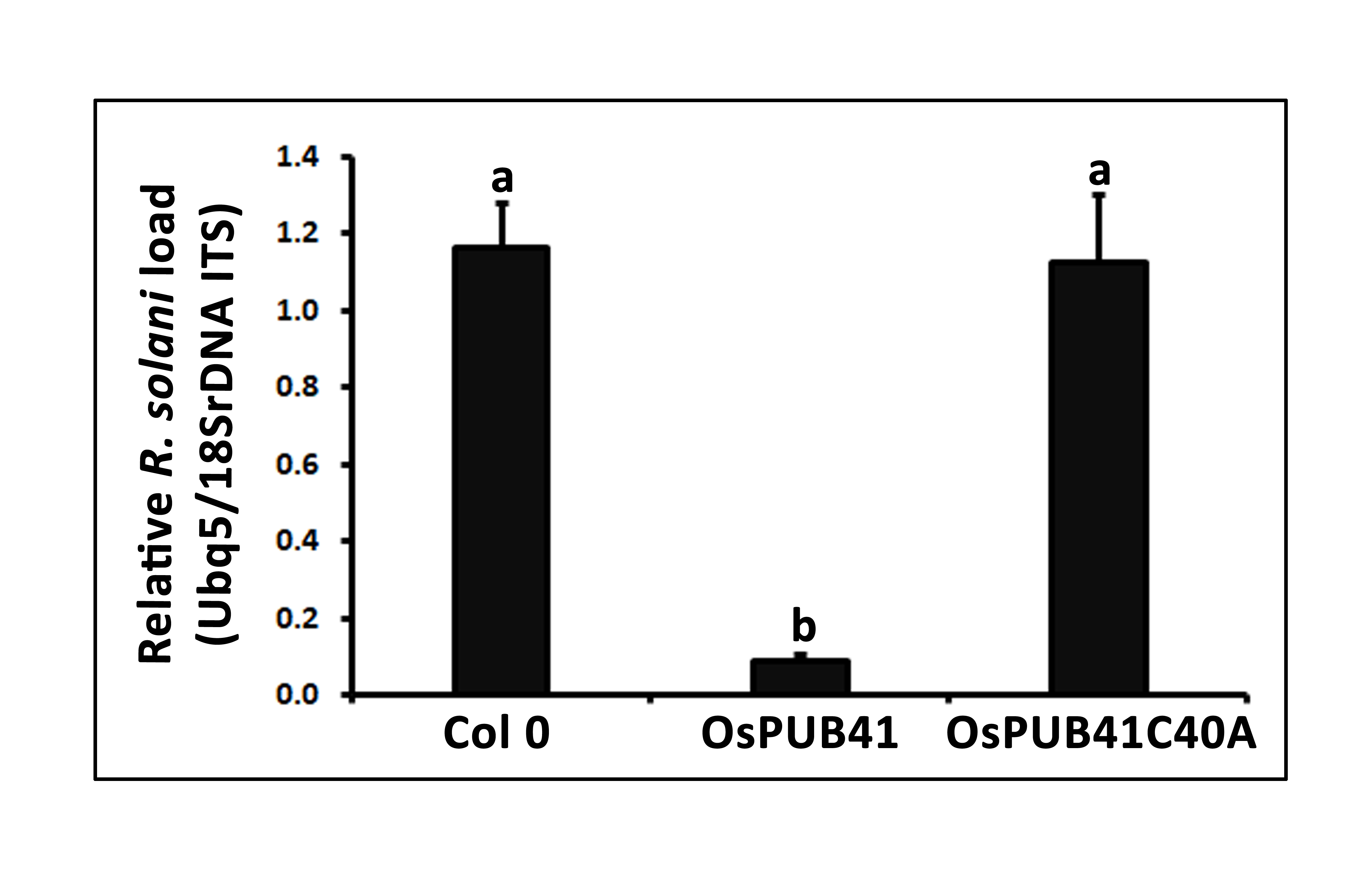

Supplement: Supplementary file 12 — Additional file 12: Fig. S4. Determination of fungal (Rhizoctonia solani AG1-1A) load during infection in Arabidopsis seedlings ectopically expressing either OsPUB41 or OsPUB41C40A. For quantitative assessment of fungal load, DNA was isolated from infected Arabidopsis seedlings (Col 0, OsPUB41 and OsPUB41C40A, 7 dpi) and used for qPCR. UBQ5F and UBQ5R (plant specific for AtUbq5 gene) and Rs1F and Rs2R (fungus specific for ITS region of 18-28S rDNA) primers (Additional file 18: Table S11) were used for qPCR. Graph represents relative level of amplification of fungal gene as compared to plant gene between induced (with Estradiol) and uninduced (with 0.1% DMSO) samples. This was calculated using the 2(−ΔΔCt) method. Three biological repeats were performed for each sample using 3 independent lines. One-way ANOVA was used to test for significance, followed by Tukey-Kramer honestly significance difference test (p < 0.05, represented by letters ‘a’ and ‘b’). [file 12870_2019_2079_MOESM12_ESM.tif]

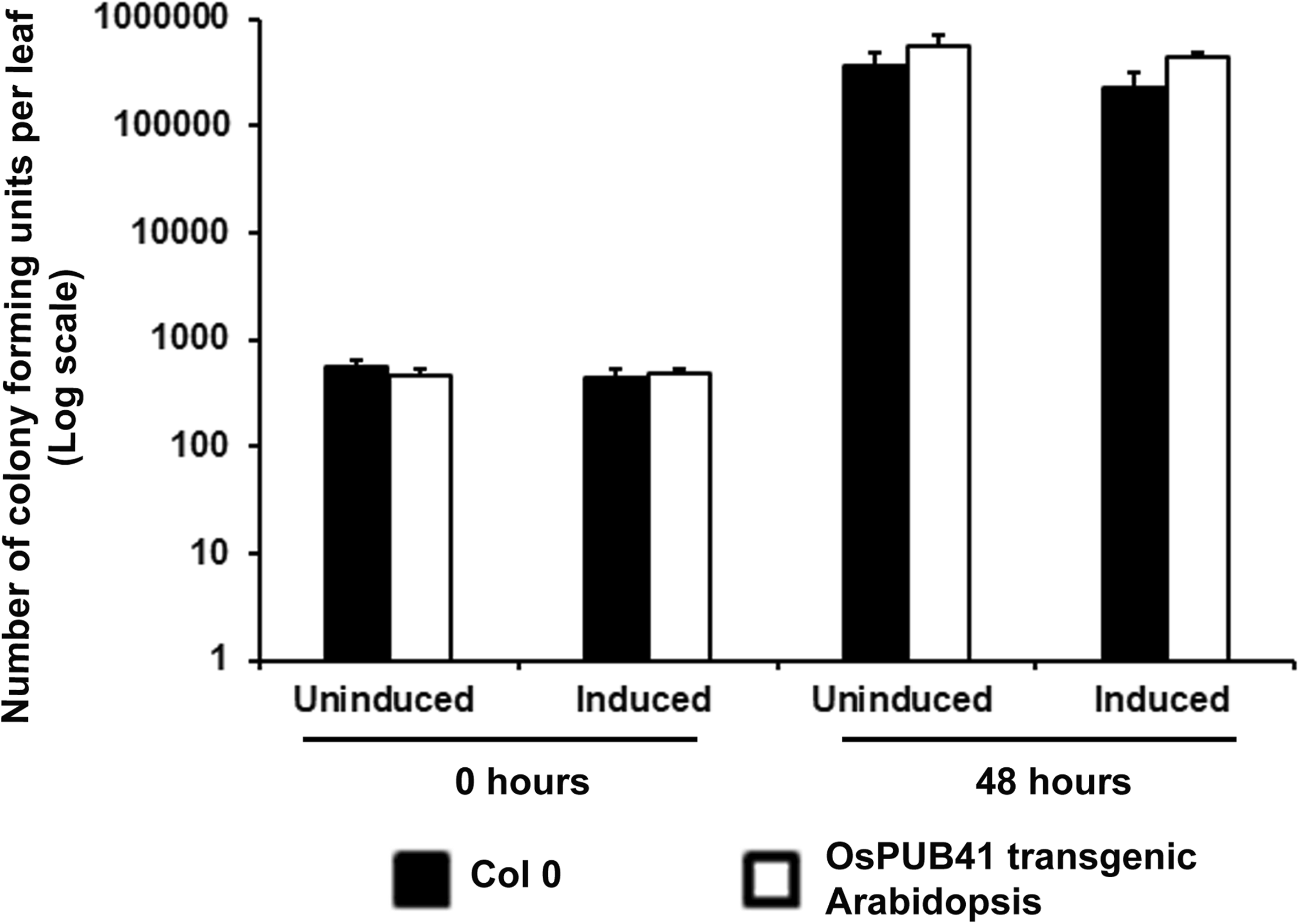

Supplement: Supplementary file 14 — Additional file 14: Fig. S5. Pst infection assay in transgenic Arabidopsis plants ectopically expressing OsPUB41. The graph represents average number of colony forming units of Pst per leaf at 0 and 48 h post infection from Col 0 and transgenic Arabidopsis plants. Y axis is logarithmic (log scale). Error bars represent standard error. Student’s two-tailed t-test for independent means was performed to test for significance (p < 0.05). Similar results were obtained in three independent experiments and in three independent transgenic lines. [file 12870_2019_2079_MOESM14_ESM.tif]

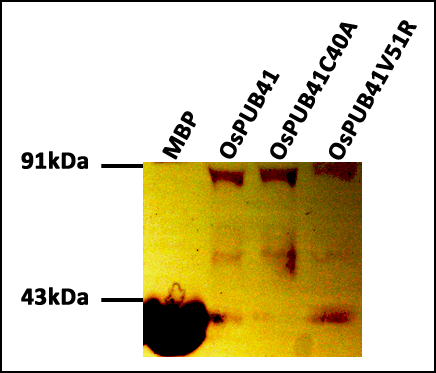

Supplement: Supplementary file 16 — Additional file 16: Fig. S6. The OsPUB41, OsPUB41C40A and OsPUB41V51R proteins were purified from E. coli. Bacterially expressed 6X-His-tagged MBP, OsPUB41, OsPUB41C40A and OsPUB41V51R proteins were purified, separated by 10% SDS-PAGE and further subjected to immunoblot analysis with anti-His antibody. Lanes 1, 2, 3 and 4 represent MBP (~ 43 kDa), OsPUB41, OsPUB41C40A and OsPUB41V51R (~ 90 kDa each) respectively. [file 12870_2019_2079_MOESM16_ESM.tif]

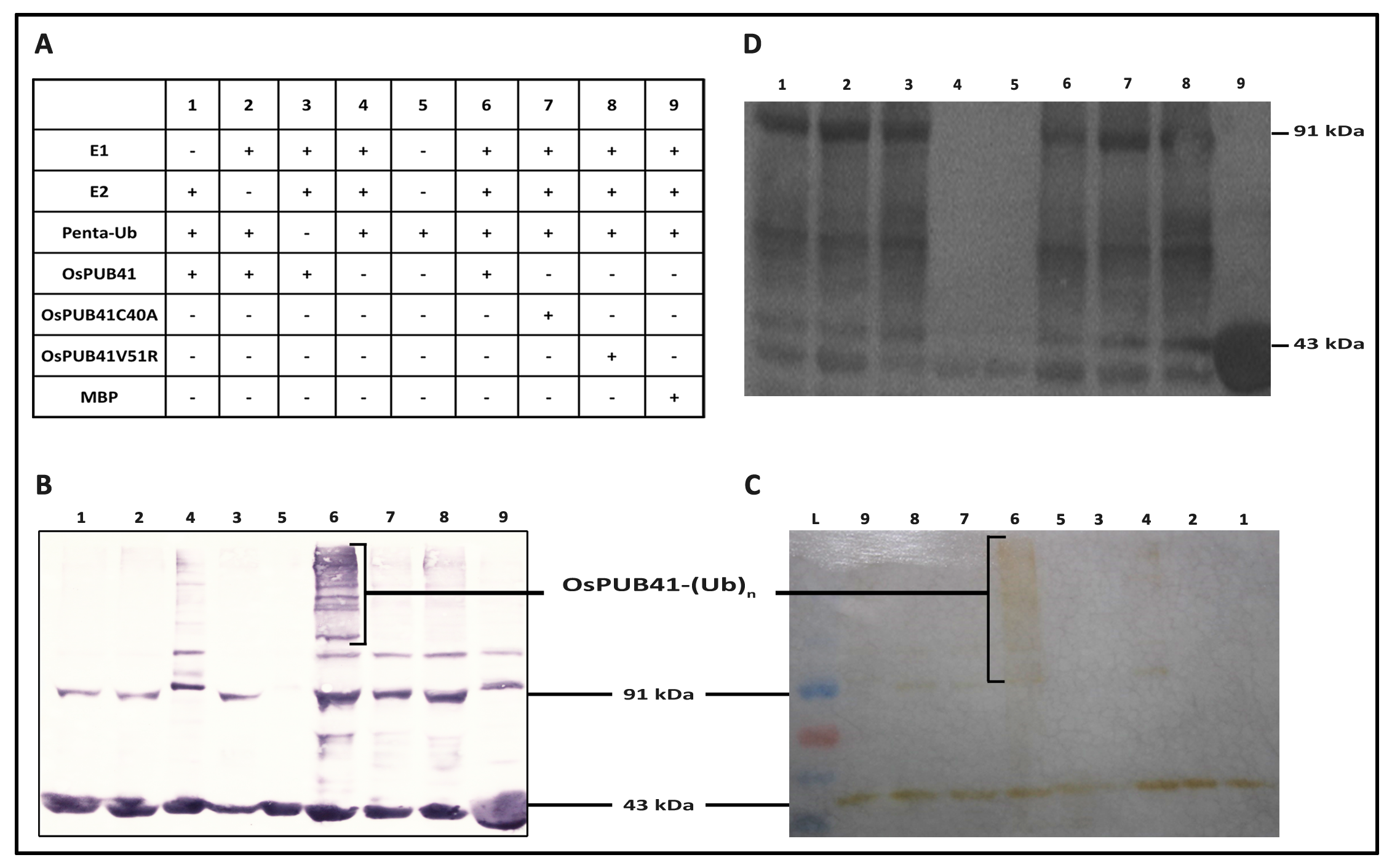

Supplement: Supplementary file 17 — Additional file 17: Fig. S7. The C40A and V51R mutations affect E3 ubiquitin ligase activity of OsPUB41. OsPUB41 protein has been shown to be a biochemically active, polyubiquitinating E3 ubiquitin ligase, by an in vitro auto-ubiquitination assay [13]. In order to generate biochemically inactive versions of OsPUB41, two independent mutants (OsPUB41C40A and OsPUB41V51R) in the U-box domain were generated. These residues (Cysteine at 40th position and Valine at 51st position) were selected because they were highly conserved across various homologues of OsPUB41 (Fig. S1B, Multiple Sequence Alignment for OsPUB41). Also it has been shown that mutation of the corresponding residues in the U-box E3 ubiquitin ligases, rice SPL11 (Valine to Arginine) and tobacco NtCMPG1 (Cysteine to Alanine), respectively, led to abolition of E3 ligase activity [19, 61]. OsPUB41 protein and its mutant forms were expressed and purified from E.coli, and tested for their E3 ligase activity using an in vitro auto-ubiquitination assay. Purified 6X-His-tagged OsPUB41 or OsPUB41C40A or OsPUB41V51R or MBP protein was incubated with ATP, 6X-His-tagged PentaUb (Ubiquitin), E1 (Ubiquitin activating enzyme; human E1) and E2 (Ubiquitin conjugating enzyme; UbcH5A) at 37 °C for four hours. The minus and the plus symbols represent absence or presence, respectively, of indicated component of the reaction mixture (A). The reaction mixtures were then resolved by 15% SDS-PAGE and subjected to immunoblot analysis with either anti-His antibody (B) or anti-Ubiquitin antibody (C) or MBP antibody (D). OsPUB41 (lane 6) protein was found to undergo polyubiquitination (bands corresponding to ≥90 kDa) whereas its mutant forms; OsPUB41C40A and OsPUB41V51R failed to exhibit E3 ligase activity (lanes 7 and 8). MBP does not affect the ubiquitination reaction (lane 9: Tag control). Lanes 3 and 4 are flipped in anti-His and anti-Ubiquitin blots as reaction mixtures loaded in these lanes (contain indicated reaction components) [file 12870_2019_2079_MOESM17_ESM.tif]
