# Supplementary material for: Overexpression of OsPUB41, a Rice E3 ubiquitin ligase induced by cell wall degrading enzymes, enhances immune responses in Rice and Arabidopsis
Source: BMC Plant Biol. 2019 Nov 29;19:530. doi: 10.1186/s12870-019-2079-1 (PMC6884774; doi:10.1186/s12870-019-2079-1)
Supplement: Supplementary file 5 — Additional file 5: Table S3. Estradiol (by itself / alone) does not induce callose deposition in rice or Arabidopsis. [file 12870_2019_2079_MOESM5_ESM.docx]

**Table S3. Estradiol (by itself / alone) does not induce callose deposition in rice or Arabidopsis**

| ^a^**Rice** | | | | |
| --- | --- | --- | --- | --- |
| ^d^Repeat | ^b^Average number of callose deposits per 0.075mm^2^ ± Standard error | | ^c^p value | Number of leaves analyzed per group (N) |
|  | TN-1 Induced | TN-1 Uninduced |  |  |
| Set 1 | 38 ± 7.2 | 34 ± 8.4 | 0.72 | 10 |
| Set 2 | 29 ± 11.5 | 32 ± 9.4 | 0.84 | 6 |
| Set 3 | 25 ± 10.4 | 30 ± 11.1 | 0.75 | 6 |
| ^e^**Arabidopsis** | | | | |
| ^h^Repeat | ^f^Average number of callose deposits per 0.075mm^2^ ± Standard error | | ^g^p value | Number of leaves analyzed per group (N) |
|  | Col-0 Uninduced | Col-0 Induced |  |  |
| Set 1 | 25 ± 5 | 29 ± 3.2 | 0.51 | 10 |
| Set 2 | 19.7 ± 6.1 | 26.1 ± 4.7 | 0.43 | 5 |
| Set 3 | 18.6 ± 4.5 | 20.8 ± 8.1 | 0.82 | 6 |

^a^Rice (TN-1) leaves were infiltrated with either DMSO (TN-1 Uninduced) or estradiol (TN-1 Induced). Twelve hours later, the leaves were stained with aniline blue and observed under an epifluorescence microscope.

^b^The tabulated value represents average number of callose deposits per field of view (0.075 mm^2^) ± standard error, from six to ten leaves (N) with six to eight different fields viewed per leaf in each experiment.

^c^Student’s two-tailed t-test for independent means was performed to test for significance (p value).

^d^Similar results were obtained in three independent experiments or repeats (Set 1, Set 2 and Set 3).

^e^Leaves of thirty-days-old Arabidopsis wild type (Col-0) plants were infiltrated either with estradiol (Col-0 Induced) or with DMSO (Col-0 Uninduced). Twelve hours later, these leaves were stained with aniline blue and observed under an epifluorescence microscope.

^f^The tabulated value represents average number of callose deposits per field of view (0.075 mm^2^) ± standard error, from five to ten leaves (N) with six to eight different fields viewed per leaf in each experiment.

^g^Student’s two-tailed t-test for independent means was performed to test for significance (p value).

^h^Similar results were obtained in three independent experiments or repeats (Set 1, Set 2 and Set 3).
