# Supplementary material for: Overexpression of OsPUB41, a Rice E3 ubiquitin ligase induced by cell wall degrading enzymes, enhances immune responses in Rice and Arabidopsis
Source: BMC Plant Biol. 2019 Nov 29;19:530. doi: 10.1186/s12870-019-2079-1 (PMC6884774; doi:10.1186/s12870-019-2079-1)
Supplement: Supplementary file 6 — Additional file 6: Table S4. Estradiol (by itself / alone) does not affect Xoo infection in rice. [file 12870_2019_2079_MOESM6_ESM.docx]

**Table S4. Estradiol (by itself / alone) does not affect Xoo infection in rice**

| ^c^Repeat | ^a^Average lesion length ± Standard error 12dpi | | ^b^p value | Number of leaves analyzed per group (N) |
| --- | --- | --- | --- | --- |
|  | TN-1 Uninduced | TN-1 Induced |  |  |
| Set 1 | 31.4 ± 6.2 | 29 ± 5.5 | 0.78 | 10 |
| Set 2 | 25 ± 5.2 | 28 ± 8.1 | 0.76 | 10 |
| Set 3 | 30 ± 5.8 | 27 ± 7 | 0.76 | 10 |

^a^Xoo infections were carried out in midveins of leaves (N = 10) of 40-days-old TN-1 rice plants. The midveins were pre-injected with DMSO (TN-1 Uninduced) or estradiol (TN-1 Induced). After 12 hours, these midveins were inoculated with Xoo (1–2 cm below the point where DMSO or estradiol was injected) by pricking with a needle dipped in a saturated Xoo (BXO43) culture. Bacterial blight lesions were measured 12 days post infection (dpi). The tabulated value represents average lesion lengths ± Standard error from ten leaves in each experiment.

^b^Student’s two-tailed t-test for independent means was performed to test for significance (p value).

^c^Similar results were obtained in three independent experiments.
