# Supplementary material for: Overexpression of OsPUB41, a Rice E3 ubiquitin ligase induced by cell wall degrading enzymes, enhances immune responses in Rice and Arabidopsis
Source: BMC Plant Biol. 2019 Nov 29;19:530. doi: 10.1186/s12870-019-2079-1 (PMC6884774; doi:10.1186/s12870-019-2079-1)
Supplement: Supplementary file 7 — Additional file 7: Table S5. Estradiol (by itself / alone) does not affect expression of defense genes in rice and Arabidopsis. [file 12870_2019_2079_MOESM7_ESM.docx]

**Table S5. Estradiol (by itself / alone) does not affect expression of defense genes in rice and Arabidopsis**

| ^a^**Rice (TN-1)** | | | | |
| --- | --- | --- | --- | --- |
| ^b^**Fold change (Induced over Uninduced) for JA biosynthetic and response genes** | | | | |
| **Gene** | **Set 1** | | **Set 2** | |
| *AOS2* | 0.90 | | 1.11 | |
| *AOC* | 1.10 | | 0.95 | |
| *LOX2* | 1.20 | | 1.20 | |
| *OPR2* | 0.88 | | 1.10 | |
| *OPR4* | 1.00 | | 1.20 | |
| *JAZ8* | 1.10 | | 0.90 | |
| *JAZ13* | 0.84 | | 0.97 | |
| ^c^**Fold change (Induced over Uninduced) for SA biosynthetic and response genes** | | | | |
| **Gene** | **Set 1** | | **Set 2** | |
| *PAL1* | 1.00 | | 1.06 | |
| *PAL2* | 1.31 | | 0.91 | |
| *SGT1* | 1.07 | | 0.89 | |
| *NH1* | 1.18 | | 1.00 | |
| *WRKY13* | 0.90 | | 1.10 | |
| ^d^**Fold change (Induced over Uninduced) for PR genes** | | | | |
| **Gene** | **Set 1** | | **Set 2** | |
| *PR1a* | 1.23 | | 0.79 | |
| *PR1b* | 1.00 | | 0.96 | |
| *PR2* | 1.12 | | 1.19 | |
| *PR3* | 0.94 | | 1.15 | |
| *PR5* | 0.86 | | 0.99 | |
| *PR9* | 1.17 | | 1.00 | |
| ^e^**Wild type Arabidopsis (Col-0)** | | | | |
| ^f^**Fold change (Induced over Uninduced) for JA biosynthetic and response genes** | | | | |
| **Gene** | **Set 1** | **Set 2** | | **Set 3** |
| *AOS* | 1.20 | 1.10 | | 0.90 |
| *PDF1.2a* | 0.88 | 1.11 | | 1.00 |
| *VSP1* | 1.06 | 1.10 | | 1.20 |
| *JAZ1* | 1.15 | 0.82 | | 0.90 |
| ^g^**Fold change (Induced over Uninduced) for SA biosynthetic and response genes** | | | | |
| **Gene** | **Set 1** | **Set 2** | | **Set 3** |
| *SID2* | 0.90 | 1.42 | | 1.00 |
| *PAL2* | 1.10 | 0.89 | | 0.90 |
| *NPR1* | 1.10 | 1.34 | | 1.22 |
| *PR1* | 1.23 | 0.86 | | 0.91 |
| *PR5* | 1.07 | 1.10 | | 0.81 |

^a^Rice leaves were infiltrated with either DMSO (Uninduced) or estradiol (Induced). Twelve hours later, these leaves were harvested and processed for qPCR analysis. Transcript levels of JA and SA biosynthetic and response genes and of *PR* genes, were measured by qPCR. *OsActin* was used as an internal control in qPCR. The table represents fold change values from two repeats. Student’s two-tailed t-test for independent means was performed on delta C_t_ values to test for significance.

^b^Jasmonic Acid (JA) biosynthetic and response genes:

*AOS2*: Allene oxide synthase2, *AOC*: Allene oxide cyclase, *LOX2*: Lipoxygenase 2 and *OPR2* and *OPR4*: 12-oxophytodienoate reductase 2 and 4 (JA biosynthetic genes)

*JAZ8* and *JAZ13*: Jasmonate ZIM-Domain 8 and 13 (JA response genes)

^c^Salicylic Acid (SA) biosynthetic and response genes:

*PAL1* and *PAL2*: Phenylalanine ammonia lyase1 and 2 (SA biosynthetic genes)

*SGT1*: SA glucosyltransferase1, *NH1*: Non-expresser of PR1 homolog1 (SA response genes), *WRKY13*: SA and JA response gene.

^d^PR genes:

*PR1a*, *PR1b*, *PR2*, *PR3*, *PR5* and *PR9*: Pathogenesis Related genes 1a, 1b, 2, 3, 5 and 9.

^e^Leaves of thirty-days-old Arabidopsis wild type (Col-0) plants were infiltrated with DMSO (Uninduced) or estradiol (Induced). Twelve hours later, these leaves were harvested and processed for qPCR analysis. Transcript levels of JA and SA biosynthetic and response genes were measured by qPCR. *AtUbq5* was used as an internal control in qPCR. The table represents fold change values from three repeats. Student’s two-tailed t-test for independent means was performed on delta C_t_ values to test for significance.

^f^Jasmonic Acid (JA) biosynthetic and response genes:

*AOS*: Allene Oxide Synthase (JA biosynthetic gene)

*PDF1.2a*: Plant Defensin, *VSP*: Vegetative Storage Protein and *JAZ*: Jasmonate ZIM-Domain (JA response genes).

^g^Salicylic Acid (SA) biosynthetic and response genes:

*SID2*: SA Induction-Deficient 2, *PAL2*: Phenylalanine Ammonia-Lyase 2 (SA biosynthetic genes)

*NPR1*: Nonexpresser of PR1, *PR1* and *PR5*: Pathogenesis Related genes 1 and 5 (SA response genes).
