# Supplementary material for: Overexpression of OsPUB41, a Rice E3 ubiquitin ligase induced by cell wall degrading enzymes, enhances immune responses in Rice and Arabidopsis
Source: BMC Plant Biol. 2019 Nov 29;19:530. doi: 10.1186/s12870-019-2079-1 (PMC6884774; doi:10.1186/s12870-019-2079-1)
Supplement: Supplementary file 9 — Additional file 9: Table S6. Callose deposition assay: Data from three transgenic Arabidopsis lines ectopically expressing either OsPUB41 or OsPUB41C40A. [file 12870_2019_2079_MOESM9_ESM.docx]

**Table S6. Callose deposition assay: Data from three transgenic Arabidopsis lines ectopically expressing either *OsPUB41* or *OsPUB41C40A***

| ^a^**Stable transgenic Arabidopsis lines ectopically expressing *OsPUB41*** | | | | |
| --- | --- | --- | --- | --- |
| **Line 33** | | | | |
| ^d^Repeat | ^b^Uninduced (Average ± Standard error) | Induced (Average ± Standard error) | ^c^p value | N (number of leaves analyzed) |
| Set 1 | 21.8 ± 3.1 | 59.2 ± 6.2 | 0.0006 | 5 |
| Set 2 | 35.5 ± 1.6 | 95.5 + 6.4 | 0.0001 | 5 |
| Set 3 | 33.1 ± 1.2 | 84 ± 7.7 | 0.0002 | 5 |
| **Line 12** | | | | |
| Repeat | Uninduced (Average ± Standard error) | Induced (Average ± Standard error) | p value | N (number of leaves analyzed) |
| Set 1 | 18 ± 4 | 55.4 ± 6.7 | 0.0014 | 5 |
| Set 2 | 23.8 ± 5.6 | 62.2 ± 10.4 | 0.0044 | 10 |
| Set 3 | 26.2 ± 4.6 | 64.8 ± 9.9 | 0.0077 | 5 |
| **Line 1** | | | | |
| Repeat | Uninduced (Average ± Standard error) | Induced (Average ± Standard error) | p value | N (number of leaves analyzed) |
| Set 1 | 29.8 ± 4.9 | 71.6 ± 9.1 | 0.0014 | 5 |
| Set 2 | 24.2 ± 10.3 | 63 ± 5.3 | 0.0102 | 5 |
| Set 3 | 16.4 ± 6.4 | 81.6 ± 5.7 | 0.0001 | 5 |
| **Stable Arabidopsis transgenic lines ectopically expressing *OsPUB41C40A*** | | | | |
| **Line 15** | | | | |
| Repeat | Uninduced (Average ± Standard error) | Induced (Average ± Standard error) | p value | N (number of leaves analyzed) |
| Set 1 | 19 ± 5.3 | 24 ± 6 | 0.55 | 5 |
| Set 2 | 25 ± 5.5 | 22.8 ± 7.1 | 0.81 | 5 |
| Set 3 | 30.2 ± 4.6 | 25.2 ± 11.1 | 0.69 | 5 |
| **Line 16** | | | | |
| Repeat | Uninduced (Average ± Standard error) | Induced (Average ± Standard error) | p value | N (number of leaves analyzed) |
| Set 1 | 26.1 ± 4.8 | 25.7 ± 0.5 | 0.94 | 5 |
| Set 2 | 31.2 ± 3.3 | 35 ± 1.9 | 0.35 | 5 |
| Set 3 | 32.4 ± 1.4 | 34.9 ± 1.8 | 0.31 | 5 |
| **Line 19** | | | | |
| Repeat | Uninduced (Average ± Standard error) | Induced (Average ± Standard error) | p value | N (number of leaves analyzed) |
| Set 1 | 30.6 ± 3.7 | 32.4 ± 6.6 | 0.82 | 5 |
| Set 2 | 34.6 ± 7.8 | 29.8 ± 4.7 | 0.61 | 5 |
| Set 3 | 28.3 ± 4.2 | 29.2 ± 3.5 | 0.88 | 6 |

^a^Leaves of thirty-days-old Arabidopsis wild type (Col-0) plants were infiltrated either with estradiol (Col-0 Induced) or with DMSO (Col-0 Uninduced). Twelve hours later, these leaves were stained with aniline blue and observed under an epifluorescence microscope.

^b^The tabulated value represents average number of callose deposits per field of view (0.075 mm^2^) ± standard error, from five to ten leaves (N) with six to eight different fields viewed per leaf in each experiment.

^c^Student’s two-tailed t-test for independent means was performed to test for significance (p value).

^d^Similar results were obtained in three independent experiments or repeats (Set 1, Set 2 and Set 3).
