# Supplementary material for: Overexpression of OsPUB41, a Rice E3 ubiquitin ligase induced by cell wall degrading enzymes, enhances immune responses in Rice and Arabidopsis
Source: BMC Plant Biol. 2019 Nov 29;19:530. doi: 10.1186/s12870-019-2079-1 (PMC6884774; doi:10.1186/s12870-019-2079-1)
Supplement: Supplementary file 10 — Additional file 10: Table S7. Ectopic expression of OsPUB41 leads to enhanced expression of Arabidopsis genes involved in JA biosynthesis and response, but does not induce SA biosynthetic and response genes: data from three transgenic Arabidopsis lines. [file 12870_2019_2079_MOESM10_ESM.docx]

**Table S7. Ectopic expression of *OsPUB41* leads to enhanced expression of Arabidopsis genes involved in JA biosynthesis and response, but does not induce SA biosynthetic and response genes: data from three transgenic Arabidopsis lines**

| ^a^**Stable Arabidopsis transgenic lines ectopically expressing *OsPUB41*** | | | | | | | | | | |
| --- | --- | --- | --- | --- | --- | --- | --- | --- | --- | --- |
| ^b^Fold change (Induced over Uninduced) for **Line 1** | | | | | | | | | | |
| Replicate | *OsPUB41* | ^c^JA biosynthetic and response genes | | | | ^d^SA biosynthetic and response genes | | | | |
|  |  | *AOS* | *PDF1.2a* | *VSP1* | *JAZ1* | *SID2* | *PAL2* | *NPR1* | *PR1* | *PR5* |
| Set 1 | 9.1 | 3.0 | 2.3 | 2.4 | 1.7 | 1.7 | 0.8 | 1.1 | 1.2 | 1.1 |
| Set 2 | 11.7 | 1.7 | 2.4 | 1.9 | 2.4 | 0.8 | 1.5 | 0.8 | 0.9 | 1.1 |
| Set 3 | 15.3 | 2.6 | 2.7 | 3.9 | 2.2 | 0.9 | 1.1 | 1.2 | 0.8 | 0.9 |
| Fold change (Induced over Uninduced) for **Line 12** | | | | | | | | | | |
| Replicate | *OsPUB41* | JA biosynthetic and response genes | | | | SA biosynthetic and response genes | | | | |
|  |  | *AOS* | *PDF1.2a* | *VSP1* | *JAZ1* | *SID2* | *PAL2* | *NPR1* | *PR1* | *PR5* |
| Set 1 | 24.2 | 3.7 | 4.3 | 4.6 | 2.0 | 1.1 | 0.9 | 0.8 | 1.1 | 0.8 |
| Set 2 | 9.4 | 3.4 | 7.0 | 2.5 | 2.3 | 0.7 | 1.4 | 1.5 | 0.8 | 1.3 |
| Set 3 | 14.3 | 2.8 | 4.0 | 3.5 | 3.2 | 1.3 | 1.0 | 1.5 | 1.3 | 1.1 |
| Fold change (Induced over Uninduced) for **Line 33** | | | | | | | | | | |
| Replicate | *OsPUB41* | JA biosynthetic and response genes | | | | SA biosynthetic and response genes | | | | |
|  |  | *AOS* | *PDF1.2a* | *VSP1* | *JAZ1* | *SID2* | *PAL2* | *NPR1* | *PR1* | *PR5* |
| Set 1 | 17.3 | 2.9 | 2.6 | 2.3 | 1.2 | 0.8 | 0.8 | 2.4 | 0.7 | 0.8 |
| Set 2 | 12.1 | 1.6 | 3.9 | 4.2 | 2.8 | 1.0 | 1.3 | 1.2 | 0.8 | 1.4 |
| Set 3 | 7.97 | 2.1 | 3.1 | 6.5 | 2.0 | 1.0 | 0.8 | 1.0 | 1.2 | 0.7 |

^a^Leaves of thirty-days-old Arabidopsis wild type (Col-0) plants were infiltrated with DMSO (Uninduced) or estradiol (Induced). Twelve hours later, these leaves were harvested and processed for qPCR analysis. Transcript levels of JA and SA biosynthetic and response genes were measured by qPCR. *AtUbq5* was used as an internal control in qPCR.

^b^The table represents fold change values from three repeats. Student’s two-tailed t-test for independent means was performed on delta C_t_ values to test for significance.

^c^Jamonic Acid (JA) biosynthetic and response genes:

*AOS*: Allene Oxide Synthase (JA biosynthetic gene)

*PDF1.2a*: Plant Defensin, *VSP*: Vegetative Storage Protein and *JAZ*: Jasmonate ZIM-Domain (JA response genes).

^d^Salicylic Acid (SA) biosynthetic and response genes:

*SID2*: SA Induction-Deficient 2, *PAL2*: Phenylalanine Ammonia-Lyase 2 (SA biosynthetic genes)

*NPR1*: Nonexpresser of PR1, *PR1* and *PR5*: Pathogenesis Related genes 1 and 5 (SA response genes).
