# Supplementary material for: Overexpression of OsPUB41, a Rice E3 ubiquitin ligase induced by cell wall degrading enzymes, enhances immune responses in Rice and Arabidopsis
Source: BMC Plant Biol. 2019 Nov 29;19:530. doi: 10.1186/s12870-019-2079-1 (PMC6884774; doi:10.1186/s12870-019-2079-1)
Supplement: Supplementary file 11 — Additional file 11: Table S8. Rhizoctonia solani AG1-1A infection assay in Arabidopsis: Data from three transgenic Arabidopsis lines ectopically expressing either OsPUB41 or OsPUB41C40A. [file 12870_2019_2079_MOESM11_ESM.docx]

**Table S8. *Rhizoctonia solani* AG1-1A infection assay in Arabidopsis: Data from three transgenic Arabidopsis lines ectopically expressing either OsPUB41 or OsPUB41C40A**

| ^a^**Wild type Arabidopsis (Col-0)** | | | | | | |
| --- | --- | --- | --- | --- | --- | --- |
|  | ^d^Set 1 | | Set 2 | | Set 3 | |
| ^b^Scoring Scale | Uninduced (Average ± Standard error) | Induced (Average ± Standard error) | Uninduced (Average ± Standard error) | Induced (Average ± Standard error) | Uninduced (Average ± Standard error) | Induced (Average ± Standard error) |
| Score 0 | 1.7 ± 1.7 | 0 ± 0 | 2.8 ± 2.1 | 1.4 ± 1.4 | 1 ± 1 | 1.2 ± 0.7 |
| Score 1 | 20.1 ± 10.5 | 20.9 ± 9.9 | 20.7 ± 2.5 | 20.8 ± 3.6 | 19.5 ± 4.3 | 20.7 ± 1.7 |
| Score 2 | 32.3 ± 8.3 | 46.7 ± 5.3 | 38.6 ± 1.1 | 37.3 ± 2.7 | 36.5 ± 3.2 | 34.3 ± 7.1 |
| Score 3 | 45.9 ± 8.6 | 32.4 ± 13.7 | 37.9 ± 2.7 | 40.6 ± 3 | 42.9 ± 5 | 43.8 ± 4.4 |
| ^c^***OsPUB41* ectopically expressing Arabidopsis transgenic Line 1** | | | | | | |
|  | Set 1 | | Set 2 | | Set 3 | |
| Scoring Scale | Uninduced (Average ± Standard error) | Induced (Average ± Standard error) | Uninduced (Average ± Standard error) | Induced (Average ± Standard error) | Uninduced (Average ± Standard error) | Induced (Average ± Standard error) |
| Score 0 | 1.8 ± 1.1 | 22.7 ± 1 | 1.1 ± 0.5 | 29 ± 4.8 | 0.8 ± 0.8 | 26.4 ± 1.3 |
| Score 1 | 10.7 ± 2.1 | 36.4 ± 2.4 | 14.3 ± 3.5 | 41.8 ± 1.8 | 21.9 ± 3.5 | 42.5 ± 2.5 |
| Score 2 | 30.4 ± 3 | 36.4 ± 2.1 | 38.9 ± 4 | 23.2 ± 3.5 | 39.7 ± 1 | 25.8 ± 0.7 |
| Score 3 | 57.1 ± 2.2 | 4.5 ± 3.1 | 45.8 ± 1.2 | 5.9 ± 1.8 | 42.7 ± 2.8 | 5.3 ± 2 |
| ***OsPUB41* ectopically expressing Arabidopsis transgenic Line 12** | | | | | | |
|  | Set 1 | | Set 2 | | Set 3 | |
| Scoring Scale | Uninduced (Average ± Standard error) | Induced (Average ± Standard error) | Uninduced (Average ± Standard error) | Induced (Average ± Standard error) | Uninduced (Average ± Standard error) | Induced (Average ± Standard error) |
| Score 0 | 1.4 ± 1.4 | 31.8 ± 5.4 | 1 ± 0.5 | 36.8 ± 4.6 | 0.9 ± 0.9 | 32.9 ± 3.5 |
| Score 1 | 12.8 ± 2.8 | 43.3 ± 2 | 11.5 ± 1.8 | 46.5 ± 1.4 | 21.2 ± 2.6 | 48.6 ± 1.8 |
| Score 2 | 41.9 ± 3.5 | 12.2 ± 2.3 | 42.2 ± 3.5 | 12.2 ± 3 | 38.5 ± 1.4 | 12.1 ± 1.6 |
| Score 3 | 44 ± 5.1 | 12.8 ± 3.1 | 45.3 ± 1.7 | 4.6 ± 2.3 | 39.4 ± 3 | 6.4 ± 3.1 |
| ***OsPUB41* ectopically expressing Arabidopsis transgenic Line 33** | | | | | | |
|  | Set 1 | | Set 2 | | Set 3 | |
| Scoring Scale | Uninduced (Average ± Standard error) | Induced (Average ± Standard error) | Uninduced (Average ± Standard error) | Induced (Average ± Standard error) | Uninduced (Average ± Standard error) | Induced (Average ± Standard error) |
| Score 0 | 1.7 ± 1.1 | 41.1 ± 3.9 | 0.7 ± 0.7 | 44.3 ± 4.2 | 1.6 ± 1.6 | 32.3 ± 4.7 |
| Score 1 | 14.4 ± 2.6 | 43.1 ± 5 | 13.3 ± 1.3 | 40.8 ± 4.5 | 15.9 ± 6.3 | 44 ± 4.9 |
| Score 2 | 37.7 ± 5.4 | 10.4 ± 2.7 | 42.6 ± 3.5 | 11.8 ± 4 | 37.6 ± 4.3 | 14.5 ± 0.2 |
| Score 3 | 46.2 ± 4.4 | 5.4 ± 1.9 | 43.4 ± 2.9 | 3.2 ± 2.8 | 44.8 ± 0.4 | 9.2 ± 0.3 |
| ***OsPUB41C40A* ectopically expressing Arabidopsis transgenic Line 15** | | | | | | |
|  | Set 1 | | Set 2 | | Set 3 | |
| Scoring Scale | Uninduced (Average ± Standard error) | Induced (Average ± Standard error) | Uninduced (Average ± Standard error) | Induced (Average ± Standard error) | Uninduced (Average ± Standard error) | Induced (Average ± Standard error) |
| Score 0 | 2 ± 0.6 | 1.3 ± 0.7 | 0 ± 0 | 0.3 ± 0.3 | 1 ± 0 | 0.7 ± 0.3 |
| Score 1 | 19.6 ± 4.5 | 20.2 ± 2.7 | 16.9 ± 3.1 | 17.7 ± 4.9 | 20 ± 2.1 | 19.7 ± 2.9 |
| Score 2 | 40.2 ± 1.4 | 39.1 ± 2.3 | 40.9 ± 2.8 | 39.7 ± 2 | 41.6 ± 1.4 | 41.6 ± 0.9 |
| Score 3 | 38.2 ± 3.7 | 39.4 ± 4.4 | 42.2 ± 4.2 | 42.3 ± 6.1 | 37.4 ± 3.3 | 38 ± 3.5 |
| ***OsPUB41C40A* ectopically expressing Arabidopsis transgenic Line 16** | | | | | | |
|  | Set 1 | | Set 2 | | Set 3 | |
| Scoring Scale | Uninduced (Average ± Standard error) | Induced (Average ± Standard error) | Uninduced (Average ± Standard error) | Induced (Average ± Standard error) | Uninduced (Average ± Standard error) | Induced (Average ± Standard error) |
| Score 0 | 2.4 ± 1.9 | 1.7 ± 1.2 | 1.3 ± 0.3 | 2 ± 0.6 | 2.3 ± 2.3 | 1.3 ± 0.6 |
| Score 1 | 16.5 ± 3.2 | 16.6 ± 2.8 | 17.7 ± 5.2 | 16.3 ± 4.3 | 18.5 ± 2.3 | 16 ± 2.1 |
| Score 2 | 40.3 ± 1.9 | 41.2 ± 2 | 42.1 ± 4.5 | 40.7 ± 5.2 | 40.1 ± 1.1 | 40.3 ± 3.5 |
| Score 3 | 40.8 ± 4.5 | 40.5 ± 1 | 38.8 ± 3.9 | 41 ± 4.3 | 39.1 ± 1.1 | 42.4 ± 1.4 |
| ***OsPUB41C40A* ectopically expressing Arabidopsis transgenic Line 19** | | | | | | |
|  | Set 1 | | Set 2 | | Set 3 | |
| Scoring Scale | Uninduced (Average ± Standard error) | Induced (Average ± Standard error) | Uninduced (Average ± Standard error) | Induced (Average ± Standard error) | Uninduced (Average ± Standard error) | Induced (Average ± Standard error) |
| Score 0 | 0.3 ± 0.3 | 1.3 ± 0.9 | 1.3 ± 0.3 | 1 ± 0 | 1.7 ± 0.3 | 1.3 ± 0.7 |
| Score 1 | 21 ± 2 | 18.7 ± 3.1 | 14.1 ± 1.9 | 17.8 ± 3.6 | 19.2 ± 1.9 | 20 ± 2.4 |
| Score 2 | 39 ± 3.2 | 40.9 ± 3.1 | 41.1 ± 2.3 | 37.4 ± 2.4 | 40.7 ± 2.1 | 38.4 ± 2.5 |
| Score 3 | 39.6 ± 3.9 | 39 ± 4.8 | 43.5 ± 3.9 | 43.8 ± 0.3 | 38.4 ± 4.2 | 40.3 ± 5 |

^a^Fifteen-days-old Arabidopsis seedlings (Col-0, *OsPUB41* and *OsPUB41C40A* transgenics) were infected with *Rhizoctonia solani* AG1-1A (*R. solani* AG1-1A), in either the presence (Induced) or absence (Uninduced) of the inducer (Estradiol) of transgene (*OsPUB41* or *OsPUB41C40A*) expression. Seven-days-post infection, the seedlings were stained with Trypan Blue and imaged using a light microscope.

^b^A scale with scores ranging from 0 to 3 was used to assess the extent of fungal infection. Score 0 = no hyphae, 1 = few unconnected hyphae, 2 = sparse continuous network of hyphae and 3 = dense network of hyphae. Values tabulated under ‘Uninduced’ and ‘Induced’ columns represent the average frequency (as a percentage) of a particular score from ten seedlings with forty different fields viewed per seedling in each experiment.

^c^Three independent transgenic lines were used.

Transgenic Arabidopsis lines ectopically expressing *OsPUB41* include lines 1, 12 and 33.

Transgenic Arabidopsis lines ectopically expressing *OsPUB41C40A* include lines 15, 16 and 19.

^d^The experiment was repeated thrice (Set 1, Set 2 and Set 3) in each transgenic line. Similar results were obtained in replicates. One-way ANOVA was used to test for significance, followed by Tukey-Kramer honestly significance difference test.
