# Supplementary material for: Overexpression of OsPUB41, a Rice E3 ubiquitin ligase induced by cell wall degrading enzymes, enhances immune responses in Rice and Arabidopsis
Source: BMC Plant Biol. 2019 Nov 29;19:530. doi: 10.1186/s12870-019-2079-1 (PMC6884774; doi:10.1186/s12870-019-2079-1)
Supplement: Supplementary file 13 — Additional file 13: Table. S9. Determination of fungal (Rhizoctonia solani AG1-1A) load during infection in Arabidopsis seedlings ectopically expressing either OsPUB41 or OsPUB41C40A: data from three transgenic Arabidopsis lines [file 12870_2019_2079_MOESM13_ESM.docx]

**Table S9. Determination of fungal (*Rhizoctonia solani* AG1-1A) load during infection in Arabidopsis seedlings ectopically expressing either *OsPUB41* or *OsPUB41C40A*: data from three transgenic Arabidopsis lines**

| ^a^**Wild type Arabidopsis (Col-0)** | | | | | | | | |
| --- | --- | --- | --- | --- | --- | --- | --- | --- |
| ^d^Repeat 1 | ^b^Fold change (Induced over uninduced) | N (Number of seedlings used) | Repeat 2 | Fold change (Induced over uninduced) | N (Number of seedlings used) | Repeat 3 | Fold change (Induced over uninduced) | N (Number of seedlings used) |
| Set1 | 1.23 | 10 | Set1 | 0.81 | 10 | Set1 | 0.87 | 10 |
| Set2 | 0.93 | 10 | Set2 | 0.76 | 10 | Set2 | 0.71 | 10 |
| Set3 | 1.32 | 10 | Set3 | 1.32 | 10 | Set3 | 1.74 | 10 |
| Average | 1.16 |  | Average | 0.96 |  | Average | 1.11 |  |
| Std error | 0.12 |  | Std error | 0.18 |  | Std error | 0.32 |  |
| ^c^***OsPUB41* ectopically expressing Arabidopsis transgenic Line 1** | | | | | | | | |
| Repeat 1 | Fold change (Induced over uninduced) | N (Number of seedlings used) | Repeat 2 | Fold change (Induced over uninduced) | N (Number of seedlings used) | Repeat 3 | Fold change (Induced over uninduced) | N (Number of seedlings used) |
| Set1 | 0.11 | 10 | Set1 | 0.47 | 10 | Set1 | 0.22 | 10 |
| Set2 | 0.23 | 10 | Set2 | 0.31 | 10 | Set2 | 0.22 | 10 |
| Set3 | 0.41 | 10 | Set3 | 0.33 | 10 | Set3 | 0.41 | 10 |
| Average | 0.25 |  | Average | 0.37 |  | Average | 0.28 |  |
| Std error | 0.09 |  | Std error | 0.05 |  | Std error | 0.06 |  |
| ***OsPUB41* ectopically expressing Arabidopsis transgenic Line 12** | | | | | | | | |
| Repeat 1 | Fold change (Induced over uninduced) | N (Number of seedlings used) | Repeat 2 | Fold change (Induced over uninduced) | N (Number of seedlings used) | Repeat 3 | Fold change (Induced over uninduced) | N (Number of seedlings used) |
| Set1 | 0.12 | 10 | Set1 | 0.18 | 10 | Set1 | 0.38 | 10 |
| Set2 | 0.14 | 10 | Set2 | 0.19 | 10 | Set2 | 0.13 | 10 |
| Set3 | 0.22 | 10 | Set3 | 0.16 | 10 | Set3 | 0.13 | 10 |
| Average | 0.16 |  | Average | 0.18 |  | Average | 0.21 |  |
| Std error | 0.03 |  | Std error | 0.01 |  | Std error | 0.08 |  |
| ***OsPUB41* ectopically expressing Arabidopsis transgenic Line 33** | | | | | | | | |
| Repeat 1 | Fold change (Induced over uninduced) | N (Number of seedlings used) | Repeat 2 | Fold change (Induced over uninduced) | N (Number of seedlings used) | Repeat 3 | Fold change (Induced over uninduced) | N (Number of seedlings used) |
| Set1 | 0.08 | 10 | Set1 | 0.09 | 10 | Set1 | 0.08 | 10 |
| Set2 | 0.12 | 10 | Set2 | 0.09 | 10 | Set2 | 0.23 | 10 |
| Set3 | 0.07 | 10 | Set3 | 0.15 | 10 | Set3 | 0.11 | 10 |
| Average | 0.09 |  | Average | 0.11 |  | Average | 0.14 |  |
| Std error | 0.01 |  | Std error | 0.02 |  | Std error | 0.05 |  |
| ***OsPUB41C40A* ectopically expressing Arabidopsis transgenic Line 15** | | | | | | | | |
| Repeat 1 | Fold change (Induced over uninduced) | N (Number of seedlings used) | Repeat 2 | Fold change (Induced over uninduced) | N (Number of seedlings used) | Repeat 3 | Fold change (Induced over uninduced) | N (Number of seedlings used) |
| Set1 | 0.81 | 10 | Set1 | 1.32 | 10 | Set1 | 1.41 | 10 |
| Set2 | 1.41 | 10 | Set2 | 0.87 | 10 | Set2 | 0.66 | 10 |
| Set3 | 1.15 | 10 | Set3 | 1.32 | 10 | Set3 | 1.23 | 10 |
| Average | 1.13 |  | Average | 1.17 |  | Average | 1.10 |  |
| Std error | 0.17 |  | Std error | 0.15 |  | Std error | 0.23 |  |
| ***OsPUB41C40A* ectopically expressing Arabidopsis transgenic Line 16** | | | | | | | | |
| Repeat 1 | Fold change (Induced over uninduced) | N (Number of seedlings used) | Repeat 2 | Fold change (Induced over uninduced) | N (Number of seedlings used) | Repeat 3 | Fold change (Induced over uninduced) | N (Number of seedlings used) |
| Set1 | 1.23 | 10 | Set1 | 1.32 | 10 | Set1 | 0.93 | 10 |
| Set2 | 0.71 | 10 | Set2 | 0.71 | 10 | Set2 | 1.15 | 10 |
| Set3 | 0.81 | 10 | Set3 | 0.87 | 10 | Set3 | 0.66 | 10 |
| Average | 0.92 |  | Average | 0.97 |  | Average | 0.91 |  |
| Std error | 0.16 |  | Std error | 0.18 |  | Std error | 0.14 |  |
| ***OsPUB41C40A* ectopically expressing Arabidopsis transgenic Line 19** | | | | | | | | |
| Repeat 1 | Fold change (Induced over uninduced) | N (Number of seedlings used) | Repeat 2 | Fold change (Induced over uninduced) | N (Number of seedlings used) | Repeat 3 | Fold change (Induced over uninduced) | N (Number of seedlings used) |
| Set1 | 0.71 | 10 | Set1 | 0.81 | 10 | Set1 | 0.62 | 10 |
| Set2 | 1.32 | 10 | Set2 | 1.32 | 10 | Set2 | 1.23 | 10 |
| Set3 | 0.76 | 10 | Set3 | 1.15 | 10 | Set3 | 1.23 | 10 |
| Average | 0.93 |  | Average | 1.09 |  | Average | 1.03 |  |
| Std error | 0.20 |  | Std error | 0.15 |  | Std error | 0.21 |  |

^a^For quantitative assessment of fungal load, DNA was isolated from infected Arabidopsis seedlings [Col-0, *OsPUB41* and *OsPUB41C40A*, 7 days post infection (dpi)] and used for qPCR. AtUBQ5F and AtUBQ5F (plant specific) and Rs1F and Rs2R (fungus specific) primers were used for qPCR.

^b^Fold change represents relative level of amplification of fungal gene as compared to plant gene between induced (with Estradiol) and uninduced (with DMSO) samples. This was calculated using the 2^(-ΔΔCt)^ method. ‘Std error’ values represent standard error.

^c^Three independent lines (for *OsPUB41* and *OsPUB41C40A*) were used for these experiments.

Transgenic Arabidopsis lines ectopically expressing *OsPUB41* include lines 1, 12 and 33.

Transgenic Arabidopsis lines ectopically expressing *OsPUB41C40A* include lines 15, 16 and 19.

^d^In each transgenic line, this experiment was repeated thrice (Set 1, Set 2 and Set 3 with 10 seedlings in each set). Three biological replicates (Repeat 1, Repeat 2 and Repeat 3) were performed (with each replicate having three sets) in each of the three transgenic lines (for OsPUB41 and for OsPUB41C40A). Student’s two-tailed t-test for independent means was performed to test for significance.
