# Supplementary material for: Overexpression of OsPUB41, a Rice E3 ubiquitin ligase induced by cell wall degrading enzymes, enhances immune responses in Rice and Arabidopsis
Source: BMC Plant Biol. 2019 Nov 29;19:530. doi: 10.1186/s12870-019-2079-1 (PMC6884774; doi:10.1186/s12870-019-2079-1)
Supplement: Supplementary file 15 — Additional file 15: Table S10. Pst infection assay in Arabidopsis: Data from three transgenic Arabidopsis lines ectopically expressing OsPUB41 [file 12870_2019_2079_MOESM15_ESM.docx]

**Table S10.** ***Pseudomonas syringae* pv. tomato DC3000 (Pst) infection assay in Arabidopsis: data from three transgenic Arabidopsis lines ectopically expressing *OsPUB41***

| **Set 1** | ^b^Average number of colony forming units (cfu) per leaf ± standard error | | | | | | | |
| --- | --- | --- | --- | --- | --- | --- | --- | --- |
|  | ^a^Wild type Arabidopsis (Col 0) | | *OsPUB41 ectopically* expressing transgenic Arabidopsis | | | | | |
|  |  |  | ^c^**Line 1** | | **Line 12** | | **Line 33** | |
| Hours post infection (hpi) | Uninduced | Induced | Uninduced | Induced | Uninduced | Induced | Uninduced | Induced |
| 0 hpi | 491 ± 70 | 480 ± 88 | 559 ± 64 | 502 ± 91 | 483 ± 95 | 522 ± 79 | 516 ± 52 | 545 ± 82 |
| 48 hpi | 201508 ± 86001 | 216916 ± 79953 | 320560 ± 94552 | 274988 ± 83410 | 230683 ± 80042 | 281499 ± 75251 | 305112 ± 58401 | 340986 ± 81940 |
| **Set 2** | Average number of colony forming units (cfu) per leaf ± standard error | | | | | | | |
|  | Wild type Arabidopsis (Col 0) | | *OsPUB41* ectopically expressing transgenic Arabidopsis | | | | | |
|  |  |  | **Line 1** | | **Line 12** | | **Line 33** | |
| Hours post infection (hpi) | Uninduced | Induced | Uninduced | Induced | Uninduced | Induced | Uninduced | Induced |
| 0 hpi | 567 ± 88 | 443 ± 88 | 494 ± 108 | 528 ± 119 | 516 ± 129 | 553 ± 103 | 457 ± 85 | 483 ± 49 |
| 48 hpi | 371667 ± 100844 | 226667 ± 90762 | 258004 ± 100061 | 295503 ± 99722 | 328611 ± 95921 | 352221 ± 119414 | 561250 ± 152847 | 437500 ± 54992 |
| **Set 3** | Average number of colony forming units (cfu) per leaf ± standard error | | | | | | | |
|  | Wild type Arabidopsis (Col 0) | | *OsPUB41* ectopically expressing transgenic Arabidopsis | | | | | |
|  |  |  | **Line 1** | | **Line 12** | | **Line 33** | |
| Hours post infection (hpi) | Uninduced | Induced | Uninduced | Induced | Uninduced | Induced | Uninduced | Induced |
| 0 hpi | 560 ± 129 | 473 ± 90 | 590 ± 88 | 523 ± 101 | 440 ± 100 | 463 ± 106 | 471 ± 94 | 483 ± 108 |
| 48 hpi | 402581 ± 96208 | 328411 ± 90526 | 351961 ± 108280 | 322017 ± 92093 | 309145 ± 69118 | 341500 ± 82301 | 319020 ± 78114 | 331905 ± 62400 |

^a^Fully expanded leaves of wild-type (Col-0) and transgenic Arabidopsis plants were pressure infiltrated with either Pst (OD_600_ of 0.02) suspended in 10mM MgCl_2_ with estradiol (Induced) or DMSO (Uninduced) using a needleless syringe into three leaves per plant. In each experiment, three plants were used per time point per condition (induced or uninduced) for each transgenic line.

^b^Bacterial growth assays were performed at 0 and 48hours post infection (hpi) to determine disease progression in the plants. Leaves were crushed in 10mM MgCl_2_ solution to acquire the bacteria. The resulting suspension was serial diluted and plated. The plates were incubated at 28°C for 48hours prior to counting of the colonies. The tabulated values represent average number of colony forming units (± Standard error) of Pst per leaf at 0 and 48hours post infection from Col-0 and transgenic Arabidopsis plants. Student’s two-tailed t-test for independent means was performed to test for significance.

^c^Similar results were obtained in three independent experiments and in three independent transgenic lines.
