# Supplementary material for: Overexpression of OsPUB41, a Rice E3 ubiquitin ligase induced by cell wall degrading enzymes, enhances immune responses in Rice and Arabidopsis
Source: BMC Plant Biol. 2019 Nov 29;19:530. doi: 10.1186/s12870-019-2079-1 (PMC6884774; doi:10.1186/s12870-019-2079-1)
Supplement: Supplementary file 18 — Additional file 18: Table S11. List of primers. [file 12870_2019_2079_MOESM18_ESM.docx]

**Table S11. List of primers**

| **Primers used for cloning and sequencing** | |
| --- | --- |
| **Name (Use)** | **Primer sequence (5’ to 3’)** |
| V51R F (SDM) | TGATGCGCGACCCGCGGACGGCGCCGACGGGGATCACGTA |
| V51R R (SDM) | TACGTGATCCCCGTCGGCGCCGTCCGCGGGTCGCGCATCA |
| C40A F (SDM) | ATCCCGGCGCACTTCCGGGCCCCGATCTCGCTGGACCTG |
| C40A R (SDM) | CAGGTCCAGCGAGATCGGGGCCCGGAAGTGCGCCGGGAT |
| OsPUB41FL F | CACCATGGCGCTGCTGGCGCGGAG |
| OsPUB41FL R | TCATGACAGCCTATTGAGCCCTCTGAAATC |
| M13F (Sequencing) | GTAAAACGACGGCCAGT |
| M13R (Sequencing) | GGAAACAGCTATGACCATG |
| pMDC7F | CAGCAGTCGAGGTAAGAT |
| pMDC7R | GGTGTGTGGGCAATGAAA |
| MBP F (Sequencing) | TACTGCGGTGATCAACGCC |
| KpnIF (Cloning) | ATTAGGGGTACCATGGCGCTGCTGGCGCGGAGG |
| Kpn1RNS (Cloning) | CCCCCGGGTACCTGACAGCCTATTGAGCCCTCTGAAATC |
| **qPCR primers used for rice genes** | |
| **Genes** | **Primer sequence (5’ to 3’)** |
| OsActin F | GAGTATGATGAGTCGGGTCCAG |
| OsActin R | ACACCAACAATCCCAAACAGAG |
| OsPUB41 F | GACCAAGGAGAAGGCCACTG |
| OsPUB41 R | ACAGCCTATTGAGCCCTCTG |
| OsAOS2RTF | CAATACGTGTACTGGTCGAATGG |
| OsAOS2RTR | AAGGTGTCGTACCGGAGGAA |
| OsAOCRTF | CGTGTACTGGTCGAATGGGC |
| OsAOCRTR | GTGAAGGTGTCGTACCGGAG |
| OsLOXRTF | GCATCCCCAACAGCACATC |
| OsLOXRTR | AATAAAGATTTGGGAGTGACATATTGG |
| OsOPR2RTF | TACACGGACTACCCGTTTCT |
| OsOPR2RTR | CGTAAGGTCGACCACAAACT |
| OsOPR4RTF | AGAGTCTCCACCAACGACTACC |
| OsOPR4RTR | CGTCCGGTGTGATCTGTATGTC |
| OsJAZ8RTF | GAAGGCTCAACAGCTGACCAT |
| OsJAZ8RTR | TTGGTGGACGGGAAGTTCTC |
| OsJAZ13RTF | CGTGAGGATGCTTATTATGCTTG |
| OsJAZ13RTR | CCAATGAAATTATATGATCCCTAGC |
| OsPAL1RTF | AGCACATCTTGGAGGGAAGCT |
| OsPAL1RTR | GCGCGGATAACCTCAATTTG |
| OsPAL2RTF | GGCCTCCACATCGCTCGC |
| OsPAL2RTR | ACGGCCTCGCGGTCGA |
| OsSGTRTF | CGCCGAGTTCCTGTCCAAGTAT |
| OsSGTRTR | ATGGCGAGGCGATCCATCAAT |
| OsNH1RTF | CACGCCTAAGCCTCGGATTA |
| OsNH1RTR | TCAGTGAGCAGCATCCTGACTAG |
| OsWRKY13RTR | TTTGGGAAAGCGTTGATTAGT |
| OsWRKY13RTF | GCGCACACACACTCCAACTC |
| OsPR1aRTF | CGTCTTCATCACCTGCAACTACTC |
| OsPR1aRTR | CATGCATAAACACGTAGCATAGCA |
| OsPR1bRTF | GGCAACTTCGTCGGACAGA |
| OsPR1bRTR | CCGTGGACCTGTTTACATTTTCA |
| OsPR2RTF | TGCTATGTTCGACGAGAACG |
| OsPR2RTR | GTTGAACAGCCCAAAGTGCT |
| OsPR3RTF | CGTGGTGACCAACATCATCA |
| OsPR3RTR | GAGTTGAAAGGCCTCTGGTTGT |
| OsPR5RTF | CAACAGCAACTACCAAGTCGTCTT |
| OsPR5RTR | CAAGGTGTCGTTTTATTCATCAAC |
| OsPR9RTF | ACGACATAAACGGGCCACAC |
| OsPR9RTR | TAGGTGCTAATGCCATGGCTG |
| **qPCR primers used for Arabidopsis genes** | |
| **Name (Locus ID)** | **Primer sequence (5’ to 3’)** |
| UBQ5 F | AAGAAGACTTACACCAAGCCGAAG |
| UBQ5 R | ACAGCGAGCTTAACCTTCTTATGC |
| AOS F | GGTGGCGAGGTTGTTTGTGATTG |
| AOS R | TTCCTAACGGCGACGTACCAAC |
| PDF 1.2a F | CTTGTTCTCTTTGCTGCTTTCGAC |
| PDF 1.2a R | TTGGCTCCTTCAAGGTTAATGCAC |
| VSP1 F | TCGAGAATCTCAAGGCTGTTGGTG |
| VSP1 R | TCAACTTCGATCCGTTTGGCTTG |
| JAZ1 F | CAGACGTGTAGTCGATTGAGTCAG |
| JAZ1 R | AAGTTCCATTGACATCAGGCTTGC |
| SID2 F | CTAATCTCCGCCGTCTCTGAACT |
| SID2 R | TTGGAACCTGTAACCGAACGA |
| PAL2 F | AGGTACTGAC AGTTACGGAG |
| PAL2 R | CATGTCTCCT TCGTGTTTCC |
| NPR1 F | TGAAGATGACGCTGCTCGATCTT |
| NPR1 R | CCCTTCATTTCGGCGATCTCCATT |
| PR1 F | GGAGCTACGCAGAACAACTAAGA |
| PR1 R | CCCACGAGGATCATAGTTGCAACTGA |
| PR5 F | CGGTACAAGTGAAGGTGCTCGTT |
| PR5 R | GCCTCGTAGATGGTTACAATGTCA |
| **qPCR primers used for *Rhizoctonia solani* AG1-IA genes** | |
| **Name** | **Primer sequence (5’ to 3’)** |
| Rs1F | GCCTTTTCTACCTTAATTTGGCAG |
| Rs2R | GTGTGTAAATTAAGTAGACAGCAAATG |
