# Supplementary material for: Overexpression of OsPUB41, a Rice E3 ubiquitin ligase induced by cell wall degrading enzymes, enhances immune responses in Rice and Arabidopsis
Source: BMC Plant Biol. 2019 Nov 29;19:530. doi: 10.1186/s12870-019-2079-1 (PMC6884774; doi:10.1186/s12870-019-2079-1)
Supplement: Supplementary file 19 — Additional file 19: Table S12. List of strains, plasmids and antibiotics [62–64]. [file 12870_2019_2079_MOESM19_ESM.docx]

**Table S12.** **List of strains, plasmids and antibiotics**

| **Strains/plasmids** | **Relevant characteristics^a^** | **Reference/source** |
| --- | --- | --- |
| ***E. coli*** | | |
| DH5α | λ– f80d*lac*ZDM15 D(*lacZYA-argF*)*U169 recA1 endA hsdR17*(rK – mK –) *supE44 thi-1 gyrA relA1* | Invitrogen |
| BL21-AI | F^-^*omp*T *hsd*S_B_ (r_B_^-^ m_B_^-^) *gal dcm ara*B::T7RNAP-*tet*A | Invitrogen |
| **Bacterial and fungal pathogens** | | |
| BXO43 (Xoo) | *rif*-2; derivative of wild type Indian isolate (BXO1) | Laboratory collection |
| *Pseudomonas syringae* pv. tomato DC3000 | Rif^r^ | (Buell *et al.*, 2003) |
| *Rhizoctonia solani* AG1-IA | - | (Ghosh *et al.*, 2014) |
| ***A. tumefaciens*** | | |
| LBA4404 | TiAch5, pAL4404;T-region, Tet^r^ , St^r^ | (Hoekema *et al.*, 1983) |
| LBA4404/pMDC7-OsPUB41 | LBA 4404/ pMDC7-OsPUB41; Tet^r^ , St^r^, Sp^r^, Hygro^r^ | This work |
| LBA4404/pMDC7- OsPUB41C40A | LBA 4404/pMDC7-OsPUB41C40A; Tet^r^, St^r^, Sp^r^, Hygro^r^ | This work |
| LBA4404/pMDC7- OsPUB41V51R | LBA 4404/pMDC7-OsPUB41V51R; Tet^r^, St^r^, Sp^r^, Hygro^r^ | This work |
| **Plasmids** | | |
| pENTR D- TOPO | Kan^r^ | Invitrogen |
| pMDC7 | 17-β-estradiol inducible binary vector, Derived from PER8 vector, Sp^r^ , Hyg^r^ | (Curtis and Grossniklaus, 2003) |
| pETM40 | Kan^r^, N-terminal MBP tag, followed by TEV protease site and C-terminal 6x His tag expression vector | G. Stier |
| pENTR-OsPUB41 | Full length *OsPUB41* in pENTR D- Topo, Kan^r^ | This work |
| pMDC7-OsPUB41 | Full length *OsPUB41* in pMDC7 T-DNA binary vector, Sp^r^ , Hyg^r^ | This work |
| MpETM40-OsPUB41 | Full length *OsPUB41* in MpETM40, Kan^r^ | This work |
| pENTR-OsPUB41C40A | *OsPUB41* with C40A mutation in pENTR D- Topo vector, Kan^r^ | This work |
| MpETM40-OsPUB41C40A | *OsPUB41* with C40A mutation in MpETM40, Kan^r^ | This work |
| pMDC7-OsPUB41C40A | *OsPUB41* with C40A mutation in pMDC7 destination vector, Sp^r^ , Hyg^r^ | This work |
| pENTR-OsPUB41V51R | *OsPUB41* with V51R mutation in pENTR D- Topo vector, Kan^r^ | This work |
| MpETM40-OsPUB41V51R | *OsPUB41* with V51R mutation in MpETM40, Kan^r^ | This work |
| pMDC7-OsPUB41V51R | *OsPUB41* with V51R mutation in pMDC7 destination vector, Sp^r^ , Hyg^r^ | This work |
| ^a^The *rif*-2 confers resistance to rifampicin; Ap^r^, Kan^r^ , Hyg^r^, Tet^r^ Sp^r^, St^r^ and Rif^r^ indicate resistance to ampicilin, kanamycin, hygromycin, tetracycline, spectinomycin, streptomycin and rifampicin respectively. | | |

Survi Mahesh (from Dr. Imran Siddiqi’s Lab) constructed MpETM40 in the following manner: pETM40 has a stop codon right after TEV-protease site. Hence, it was digested with NcoI and XhoI and then that part was replaced with the following 41bp oligo, containing a TEV-protease site: ACGGTACCGGATCCGAATTCGAGAATCTTTATTTTCAGGGC (6,251-6,291), regenerating NcoI and XhoI restriction sites.
